# Untargeted serum metabolomics reveals novel metabolite associations and disruptions in amino acid and lipid metabolism in Parkinson’s disease

**DOI:** 10.1186/s13024-023-00694-5

**Published:** 2023-12-19

**Authors:** Kimberly C. Paul, Keren Zhang, Douglas I. Walker, Janet Sinsheimer, Yu Yu, Cynthia Kusters, Irish Del Rosario, Aline Duarte Folle, Adrienne M. Keener, Jeff Bronstein, Dean P. Jones, Beate Ritz

**Affiliations:** 1 Department of Neurology, UCLA David Geffen School of Medicine, Los Angeles, CA USA; 2 Department of Epidemiology, UCLA Fielding School of Public Health, Los Angeles, CA USA; 3 Gangarosa Department of Environmental Health, Rollins School of Public Health, Emory University, Atlanta, GA USA; 4 Department of Human Genetics, UCLA David Geffen School of Medicine, Los Angeles, CA USA; 5 Department of Biostatistics, UCLA Fielding School of Public Health, Los Angeles, CA USA; 6 Center for Health Policy Research, UCLA Fielding School of Public Health, Los Angeles, CA USA; 7 Parkinson’s Disease Research, Education, and Clinical Center, Greater Los Angeles Veterans Affairs Medical Center, Los Angeles, CA USA; 8 Department of Medicine, School of Medicine, Emory University, Atlanta, GA USA

## Abstract

**Background:**

Untargeted high-resolution metabolomic profiling provides simultaneous measurement of thousands of metabolites. Metabolic networks based on these data can help uncover disease-related perturbations across interconnected pathways.

**Objective:**

Identify metabolic disturbances associated with Parkinson’s disease (PD) in two population-based studies using untargeted metabolomics.

**Methods:**

We performed a metabolome-wide association study (MWAS) of PD using serum-based untargeted metabolomics data derived from liquid chromatography with high-resolution mass spectrometry (LC-HRMS) using two distinct population-based case-control populations. We also combined our results with a previous publication of 34 metabolites linked to PD in a large-scale, untargeted MWAS to assess external validation.

**Results:**

LC-HRMS detected 4,762 metabolites for analysis (HILIC: 2716 metabolites; C18: 2046 metabolites). We identified 296 features associated with PD at FDR<0.05, 134 having a log_2_ fold change (FC) beyond ±0.5 (228 beyond ±0.25). Of these, 104 were independently associated with PD in both discovery and replication studies at *p*<0.05 (170 at *p*<0.10), while 27 were associated with levodopa-equivalent dose among the PD patients. Intriguingly, among the externally validated features were the microbial-related metabolites, p-cresol glucuronide (FC=2.52, 95% CI=1.67, 3.81, FDR=7.8e-04) and p-cresol sulfate. P-cresol glucuronide was also associated with motor symptoms among patients. Additional externally validated metabolites associated with PD include phenylacetyl-L-glutamine, trigonelline, kynurenine, biliverdin, and pantothenic acid. Novel associations include the anti-inflammatory metabolite itaconate (FC=0.79, 95% CI=0.73, 0.86; FDR=2.17E-06) and cysteine-S-sulfate (FC=1.56, 95% CI=1.39, 1.75; FDR=3.43E-11). Seventeen pathways were enriched, including several related to amino acid and lipid metabolism.

**Conclusions:**

Our results revealed PD-associated metabolites, confirming several previous observations, including for p-cresol glucuronide, and newly implicating interesting metabolites, such as itaconate. Our data also suggests metabolic disturbances in amino acid and lipid metabolism and inflammatory processes in PD.

**Supplementary Information:**

The online version contains supplementary material available at 10.1186/s13024-023-00694-5.

## Introduction

Parkinson’s disease (PD) is a complex, multi-factorial neurodegenerative disease with multi-system involvement. Pathologically, PD is defined by the loss of dopaminergic neurons in the substantia nigra and widespread intracytoplasmic aggregations of misfolded α-synuclein [1]. Rare genetic mutations have been identified in early-onset, familial PD, but idiopathic PD’s complex etiopathogenesis remains unclear [2].

High-throughput technological developments over the past decade have paved the way for agnostic analysis of multiple omic measures, providing novel insight into disease etiology. Genome-wide association studies (GWAS), for instance, have highlighted the role of endolysosomal (vesicle trafficking, lysosomes, and autophagy) and immune pathways in PD [3]. Still, biologic processes are dynamic and operate through complex interactions between gene expression, protein function, and metabolism. Investigating other principal omics, including the metabolome, may provide new insights into biologic processes involved in PD.

Recent advances in high-resolution metabolomic profiling allow for the simultaneous measurement of thousands of metabolites. Metabolic networks shed light on the underlying biochemical activity of cells, tissues, and organs, enabling a multi-system level approach to the study of PD. Furthermore, metabolites reflect the convergence of genomic, epigenomic, transcriptomic, and proteomic action in tandem with the system’s response to environmental exposures, thus offering a readout of both physiologic and pathologic states of an individual [4]. Metabolites circulating in the blood provide a wealth of information about biologic processes across different systems, including the central nervous system as metabolites can cross the blood-brain barrier [5]. A growing body of work supports the use of metabolomics to provide novel information about the initiation and progression of PD [5–7]. For instance, metabolites related to lipid metabolism, including glycerophospholipids and sphingolipids, mitochondrial function, and amino acids have been implicated in PD [8, 9].

Here, we have performed a series of untargeted metabolome-wide association studies (MWAS) to explore serum metabolite signatures associated with PD. Using metabolite profiles measured by a dual-column, dual-polarity liquid-chromatography approach with high-resolution mass spectrometry (LC-HRMS), we performed an MWAS with independent discovery and replication study populations. We identified individual metabolite features associated with PD, evaluated pathway enrichment, and investigated associations between PD-MWAS metabolites and symptom profiles among patients. We assessed both replication of the metabolite findings, using the two similar but independent study populations from California, as well as external validation of metabolites previously associated with PD in a metabolomic profiling of drug-naïve patients from a hospital-based study of Chinese PD patients and healthy controls [7]. This previous study analyzed 226 metabolites and associated 50 with PD. We build on this work, analyzing nearly 5000 metabolite features using two community-based studies of PD.

Ultimately, identifying disrupted metabolic pathways in PD may improve our understanding of the molecular mechanisms underlying pathogenesis, paving the way for new preventative or therapeutic strategies.

## Methods

### Study population

We used metabolomic profiles from 642 PD patients and 277 controls recruited as part of a community-based study of Parkinson’s disease (Parkinson’s Environment and Genes study, PEG). PEG is a population-based PD case-control study conducted in three Central California counties [10]. Participants were recruited in two, independent study waves: PEG1, 2000-2007 and PEG2, 2011-2018. All those with serum for metabolomics were included (PEG1: *n*=282 PD patients, *n*=185 controls; PEG2: *n*=360 PD patients, *n*=90 controls). Patients were early in disease course at enrollment (3.0 years [SD=2.6] on average from diagnosis) and all were seen by UCLA movement disorder specialists for in-person neurologic exams and confirmed as having idiopathic PD based on clinical characteristics [11]. Characteristics of the PEG study subjects are shown in Supplemental Table 1. The patients were on average slightly older than the controls and a higher proportion of the patients were men, Hispanic, and never smokers compared to the controls.

### Sample collection

Blood samples were drawn from participants during field visits. Samples were centrifuged, kept on dry ice, and then stored in a −80 °C freezer at UCLA. Serum samples were shipped frozen to Emory University on dry ice for metabolomics analyses, where they were stored at −80 °C until analyses.

### High-Resolution Metabolomics (HRM)

HRM profiling was conducted according to established methods. Detailed methods are provided in previous publication [12]. Briefly, serum samples were randomly sorted into batches of 40. Each sample was thoroughly mixed with ice-cold acetonitrile (2:1 acetonitrile to serum), placed on ice for 30 minutes, precipitated protein was removed by centrifugation, and the resulting supernatant was transferred to an autosampler vial containing a low volume insert. We analyzed all sample extracts in triplicate with a dual-column, dual-polarity approach, including hydrophilic interaction (HILIC) chromatography with positive electrospray ionization (ESI) and C18 chromatography with negative ESI, and used two types of quality control samples. We included two methods of performance quality control. First, a NIST 1950 QC sample was analyzed at the beginning and end of the entire analytical run [13]. A second QC sample (Q-Std), which is commercially purchased plasma pooled from an unknown number of men and women, was analyzed at the beginning, middle, and end of each batch of 40 samples for normalization and batch effect evaluation (*n*=180 Q-Std samples total included).

The Emory metabolomics lab uses a quality control procedure based on XCMS and a set of confirmed metabolites and internal standards to evaluate the data quality of each batch: number of features detected, missing values, mass accuracy (threshold <5 ppm), Pearson correlation within technical replicates (threshold: 0.9), and average coefficient of variation (CV) of feature intensities within replicates (threshold: <30%). Samples were re-analyzed if the data did not meet the defined criteria.

Our samples were processed across two LC-HRMS runs conducted approximately 6-months apart, to pool the metabolite data across runs, we used the *apLCMS* R package to perform retention time adjustment and feature alignment for both HILIC and C18 feature tables, using the adjust.time and feature.align functions [14]. For feature alignment, the *m*/*z* tolerance was 1e-05 and retention time tolerance was 37.016 (C18) and 38.246 (HILIC) seconds. Overall, 2226 features aligned for C18 and 2919 for HILIC across the two LCMS runs. For analyses, we included metabolomic features with median CV among technical replicates <30% and Pearson correlation >0.9 and features detected in >50% of all study samples, leaving 2046 C18 features and 2716 HILIC features for analysis.

We log 2 transformed the metabolite data, quantile normalized, and batch corrected with ComBat after replacing zeroes with the lowest detected value which has been recommended for metabolomics data. Data pre-processing visualization is shown in Supplemental Figs. 1, 2, 3 and 4. From principal component (PC) analysis with the HILIC features, we discovered two clusters of samples seemingly separating based on technical, non-biologic factors. As a result, we performed an additional correction to remove variation between the PCs (Supplemental Figs. 5, 6 and 7). This was done with ComBat, using an indicator for whether the sample was part of the outlying cluster as the correction term.

Within the Q-Std samples across both runs and all batches (*n*=180), the mean CV across all C18 metabolite features before the data processing steps was 157.1% (median=75.2%, IQR=127.1%) but after the processing steps it reduced to 7.2% (median=6.3%, IQR=5.5%). For HILIC features, the mean CV before processing was 148.0% (median=69.3%, IQR=128.0%) and after the processing steps 8.7% (median=8.0%, IQR=8.3%).

### Metabolome-Wide Association Analysis (MWAS)

To identify metabolite features associated with PD, we conducted two sets of MWAS analyses. First, we fit a linear regression model, using the *limma* R package and empirical Bayes (eBayes) function [15], providing a log2 fold change (log_2_FC) estimate comparing patients and controls. Second, we used unconditional logistic regression for each metabolite with PD as the outcome to provide odds ratio estimates. We determined metabolite associations independently for the PEG1 and PEG2 case-control studies and then combined odds ratio (OR) estimates in a fixed effects meta-analysis, using a generic inverse-variance method for pooling [16]. For both analyses, we controlled for age, gender, race/ethnicity, a year of sample draw indicator, and study wave as covariates. We used a false discovery rate (FDR) to correct for multiple testing. We prioritized metabolites based on significance (FDR<0.05) and log_2_FC thresholds at ±0.5 (higher-level of importance) and ±0.25 (lower-level).

We assessed replication [17], meaning confirmation of associations across the independent, but similar PEG1 and PEG2 study populations, which are from the same communities, recruited some 10-years apart, with overlapping study design, data collection and identical laboratory methods. Replication was based on independent association of metabolites in both discovery (PEG1) and replication (PEG2) populations at *p*<0.05 and log_2_FC at least ±0.25.

For metabolites which showed association with PD, we further tested for association with the following PD and PD symptom related phenotypes among PD patients only using linear regression: levodopa equivalent daily dose (LEDD), Hoehn Yahr (HY) stage, and Unified Parkinson’s disease Rating Scale Part III (UPDRS-III) score.

We performed age-related sensitivity analyses for p-cresol metabolites as these metabolites showed positive correlation with age among both patients and controls. Within the study population, we matched patients to controls based on age (±2 years), gender, and race, as a 1:1 match and a 2:1 match, then we assessed metabolite associations using the matched data only. In sensitivity analyses, we also processed and analyzed each HRMS runs independently using the same processing pipeline and *limma* to calculate log_2_FC estimates by batch.

### Annotation and pathway analysis

We annotated features based on three levels. First, significant features were matched to a database of authenticated chemical standards previously characterized in the Emory laboratory, i.e., metabolites confirmed using MS/MS and authentic standards, providing the strongest level of annotation [18, 19]. The error tolerance was set to 5 ppm and 30s for *m*/*z* and retention time. Additional *m*/*z* feature mapping was done based on *mummichog* annotations and *xMSannotator*. *mummichog* is a computational algorithm which uses metabolic pathways and networks to predict functional activity from untargeted metabolite feature tables, including providing annotations of features based upon predicted ions and pathway associations [20]. With *xMSannotator*, accurate mass *m*/*z* for adducts formed under positive/negative ESI mode were matched to HMDB, KEGG, and LipidMaps with a mass error threshold of 10 ppm [21]. *xMSannotator* uses correlations of intensities and retention time and assigns confidence scores based on a multilevel scoring algorithm (0–3, a higher score representing higher-confidence result), ensuring annotation accuracy. Only results with scores ≥2 were considered for annotations.

For pathway enrichment analysis we used *metapone*, which uses a permutation-based weighted hypergeometric test with joint pathway analysis using positive and negative ion mode data to avoid double counting and account for multiple-matching uncertainty with a weighting factor [22]. Metabolic pathways were compiled from KEGG, *mummichog*, and the small molecule pathway database (SMPDB).

### External validation and meta-analysis

For external validation, meaning confirmation of metabolite associations in a different population [17], we compared our results to a previous report of untargeted metabolomics in PD [7]. The two studies were different with regard to study location (China and United States), racial composition, recruitment (hospital-based and community-based), PD medication status (drug-naïve and L-dopa medicated), and other lifestyle and exposure factors such as diet. Given such differences, in general, large-scale, agnostic omics studies that achieve reproducibility based on external validity indicate a robust association [17].

The previous report detailed an untargeted metabolic profiling of PD from a Chinese population comparing drug-naïve patients recruited from a hospital to healthy controls (*n*=223 PD and *n*=237 controls). They measured 226 metabolites with LCMS, limiting analysis to metabolites identified using internal standards. Overall, 50 were associated with PD [17]. In the current study, we detected 34 of the 50 metabolites in our sample, identified through either the Emory metabolomics LCMS in house library or high confidence annotation in with *xmsAnnotator*. As only the fold changes, *p*-values, and sample size were available from the previous study, we used two R packages designed to combine fold changes and *p*-values across studies. First, the *amanida* package, which combines *p*-values from the individual studies using Fisher’s method and fold-changes by averaging, with both weighted by the study size [23]. Second, we used *metaDEA*, which similarly averages the study-specific log_2_FC, but also calculates the SD, and estimates a “pseudo t-score” (the ratio of the mean log_2_FC over the SD of the log_2_FCs divided by the square root of the number of comparisons) [24]. The absolute value of this score is higher for metabolites with similar log_2_FCs across the two studies, thus prioritizing consistency of the estimates. Validation was assessed at three levels: (1) significance, with the combined adj *p*-value<0.05 and the individual studies having a *p*<0.25; (2) direction of effect; and (3) magnitude, with a combined log_2_FC threshold of ±0.25.

## Results

### Metabolome wide association study

**Fig. 1:**
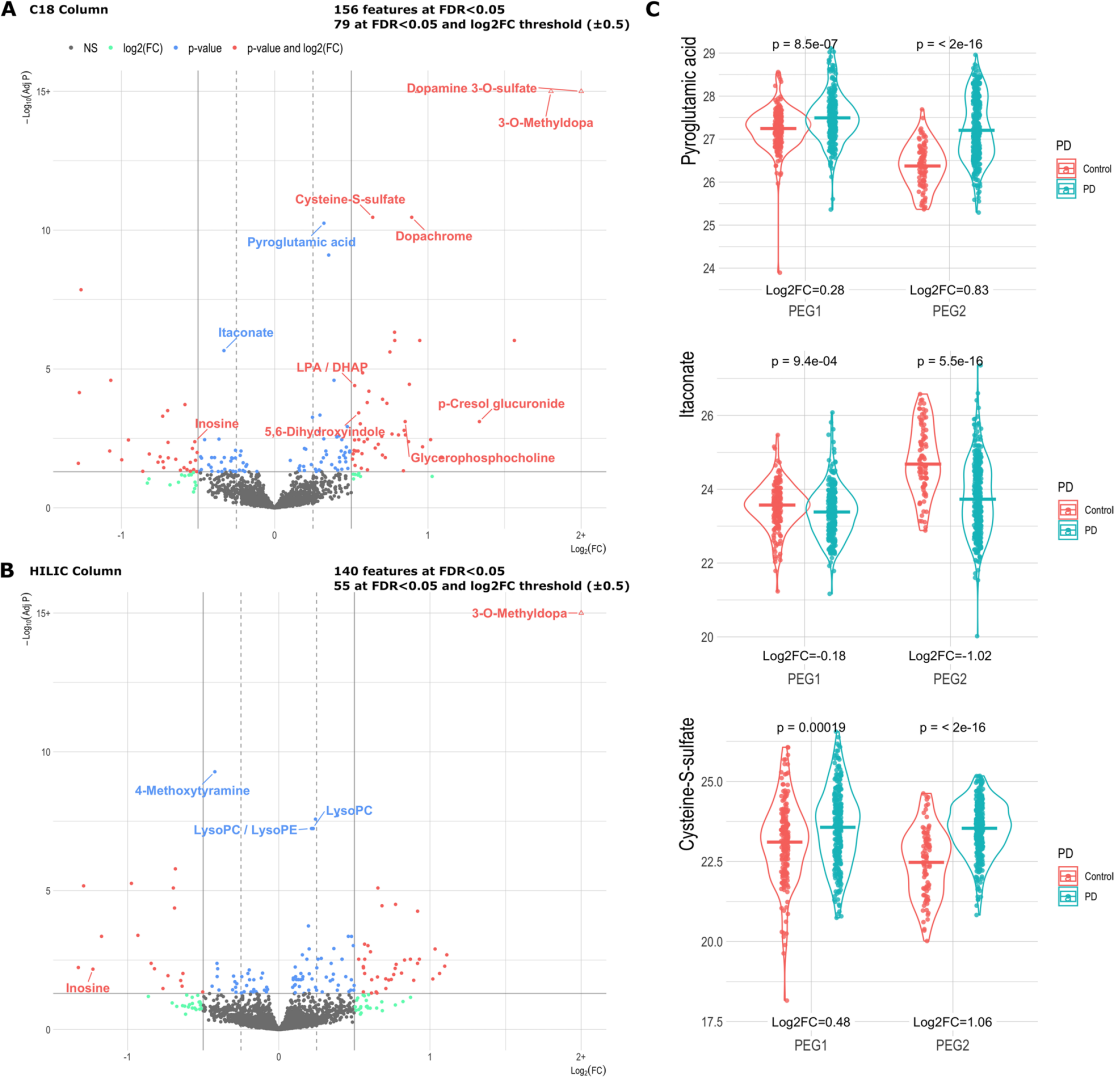
Volcano Plot of the MWAS results from metabolomics LCMS data derived from both the (**A**) C18 negative and (**B**) HILIC positive results. Horizontal lines are shown at FDR≤0.05 and vertical lines are shown at log_2_FC ±0.5 and dashed lines at log_2_FC ±0.25. Metabolite features with a -log_10_(FDR)>15 and/or log_2_FC>2 are designated by the triangle shape and shown at the -log_10_(FDR)=15 and or log_2_FC =2 lines. Exact results can be found in Supplemental Tables 2 (C18 metabolites) and 3 (HILIC metabolites). **C** Violin plots show the top three metabolites from the MWAS by FDR that could be annotated at the highest confidence, excluding PD medication-associated metabolites, and separated by the independent study populations.

Our untargeted metabolome-wide association study included 4762 features for analysis (2046 C18 and 2716 HILIC). Overall, based on the linear model fit with *limma* and eBayes, 296 metabolite features (156 on C18; 140 on HILIC) showed evidence of differential abundance between patients and controls (FDR<0.05), with 134 (79 on C18; 55 on HILIC) having a log_2_FC beyond the ±0.5 threshold (228 beyond ±0.25). Fig. 1 A and B show volcano plots of the MWAS results. The full MWAS summary statistics are provided in Supplemental Table 2 (C18 metabolites) and Supplemental Table 3 (HILIC metabolites). We also show results from the logistic regression model of PD risk by metabolite feature in the same supplemental tables and for analyses processed and stratified by HRMS run (Supplemental Tables 2 and 3 and Supplemental Figs. 8, 9, 10 and 11).

Of the associated metabolite features, 104 met our criteria for independent replication between our studies (discovery and replication *p*<0.05), with 50 showing a log_2_FC beyond ±0.5 (86 beyond ±0.25). From the C18 column, 66 features were independently associated in discovery and replication at *p*<0.05 (116 at *p*<0.10), with 41 having a log_2_FC beyond the ±0.5 (63 beyond ±0.25). From the HILIC column, 38 of the features were associated in discovery and replication at *p*<0.05 (54 at *p*<0.10), with 9 having a log_2_FC beyond ±0.5 (23 beyond ±0.25).

Annotation based on three-layers (in-house database of metabolites, *mummichog* annotations, and *xMSannotator* high confidence matches) for all features with discovery and replication at *p*<0.1 is provided in Supplemental Table 4 (C18) and Supplemental Table 5 (HILIC). The full *xMSannotator* stage 5 annotation results are provided in Supplemental Table 6 (C18) and Supplemental Table 7 (HILIC).

Table 1 shows the top MWAS metabolites that were associated with PD in discovery and replication cohorts. As expected, the leading PD-associated features from both columns were related to PD-medications, including medication metabolites, dopamine 3-O-sulfate and 3-O-methyldopa. In total, from the 4762 metabolite features for analysis, 11 C18 features and 16 HILIC features were associated with LEDD at an FDR<0.05 among the PD patients (Supplemental Tables 2 and 3). The majority of PD-associated metabolites were not strongly associated with levodopa medication use.

**Table 1:** PD MWAS hits: Annotated features from the MWAS associated with Parkinson’s disease in both discovery and replication study populations

**mz**	**time**	**Fold Change (95% CI)**	**FDR**	**Meta OR (95% CI)**	**Meta FDR**	**Discovery (PEG1)**	**Replication (PEG2)**	**LEDD *p*-value**	**LEDD FDR**	Compound Annotation*
**C18 Negative**										
232.0282	34.8165	13.85 (8.73, 21.97)	3.27E-24	1.24 (1.18, 1.29)	1.71E-17	1.05E-16	1.15E-05	8.70E-37	8.90E-34	Dopamine 3-O-sulfate^2,3^
210.0772	35.9778	3.49 (2.81, 4.34)	3.27E-24	1.72 (1.53, 1.94)	1.09E-16	4.49E-15	2.33E-07	3.17E-76	6.48E-73	3-O-Methyldopa^1,2,3^
128.0353	30.6303	1.25 (1.18, 1.32)	5.61E-11	3.79 (2.60, 5.54)	3.51E-09	4.86E-07	1.42E-08	1.84E-01	7.09E-01	Pyroglutamic acid/ Pyrrolidonecarboxylic acid^1,3^
192.0305	24.1011	1.86 (1.58, 2.18)	3.43E-11	1.55 (1.36, 1.76)	1.81E-08	7.84E-10	4.42E-03	2.44E-26	1.67E-23	Dopachrome^2,3^
129.0192	33.8177	0.79 (0.73, 0.86)	2.17E-06	0.47 (0.36, 0.61)	7.32E-06	6.14E-04	9.36E-07	5.10E-02	5.24E-01	Itaconate (Itaconic acid)^1,3^
435.2514	167.2708	1.44 (1.25, 1.65)	3.94E-05	1.62 (1.36, 1.92)	8.20E-06	1.01E-04	8.85E-05	1.09E-02	3.42E-01	LPA(0:018:1(9Z)); LPA(18:1(9Z)0:0); DHAP(18:0)^3^
199.9693	28.4295	1.56 (1.39, 1.75)	3.43E-11	1.56 (1.32, 1.83)	1.49E-05	7.10E-05	2.95E-10	1.29E-01	6.33E-01	Cysteine-S-sulfate^3^
145.0143	28.1639	1.22 (1.11, 1.35)	1.75E-03	1.61 (1.31, 1.98)	7.78E-04	9.43E-04	2.79E-04	5.46E-02	5.32E-01	Oxoglutaric acid^1,2,3^
146.0459	29.4772	1.19 (1.10, 1.28)	5.47E-04	1.86 (1.40, 2.46)	1.26E-03	2.98E-03	4.24E-04	7.60E-02	5.94E-01	Glutamic acid^2,3^
283.0821	30.1765	2.52 (1.67, 3.81)	7.82E-04	1.10 (1.05, 1.15)	3.56E-03	3.63E-04	5.57E-02	8.07E-01	9.66E-01	p-Cresol glucuronide^3^
256.0936	28.4837	1.80 (1.39, 2.35)	7.82E-04	1.14 (1.06, 1.22)	7.97E-03	4.35E-03	2.03E-05	7.59E-02	5.94E-01	Glycerophosphocholine^3^
107.0502	34.5396	1.32 (1.16, 1.52)	2.80E-03	1.29 (1.13, 1.47)	1.08E-02	5.43E-03	7.76E-03	2.44E-01	7.52E-01	p-Cresol^3^
267.0723	32.8807	0.70 (0.58, 0.84)	4.15E-03	0.82 (0.74, 0.92)	1.36E-02	1.67E-02	3.13E-05	2.85E-02	4.51E-01	Inosine^2,3^
148.0405	31.5075	1.46 (1.24, 1.72)	3.80E-04	1.27 (1.12, 1.45)	1.41E-02	1.12E-02	2.23E-03	4.38E-20	1.49E-17	5,6-Dihydroxyindole^2,3^
187.0071	35.7939	1.35 (1.17, 1.57)	3.50E-03	1.24 (1.10, 1.39)	1.77E-02	1.03E-02	1.06E-02	3.46E-01	8.40E-01	p-Cresol sulfate^3^
151.0514	33.6125	0.70 (0.58, 0.85)	1.01E-04	0.84 (0.77, 0.93)	2.21E-02	8.23E-03	2.32E-02	5.50E-01	9.03E-01	N1-Methyl-4-pyridone-5-carboxamide^3^
172.9914	34.1845	0.79 (0.67, 0.93)	6.08E-02	0.83 (0.74, 0.93)	3.21E-02	4.29E-02	4.95E-03	3.51E-01	8.42E-01	Phenol sulphate^3^
299.2014	200.4142	1.46 (1.19, 1.79)	8.99E-03	1.16 (1.06, 1.26)	3.34E-02	8.45E-02	1.82E-04	1.21E-02	3.51E-01	all-trans-Retinoic acid^1,3^
189.0029	34.3209	1.40 (1.17, 1.69)	8.99E-03	1.17 (1.06, 1.29)	3.98E-02	3.58E-02	1.26E-02	2.64E-01	7.82E-01	Oxalosuccinate^2,3^
**HILIC Positive**										
212.0917	60.9225	35.1 (23.0, 53.4)	1.51E-50	1.43 (1.35, 1.52)	4.04E-31	1.08E-28	5.49E-08	3.32E-70	9.03E-67	3-O-Methyldopa^3^
185.1285	46.3928	0.75 (0.69, 0.81)	5.21E-10	0.42 (0.32, 0.54)	3.05E-08	1.34E-07	3.79E-05	2.37E-07	1.61E-04	4-Methoxytyramine^3^
510.3557	47.829	1.16 (1.11, 1.22)	5.77E-08	4.06 (2.47, 6.65)	1.59E-05	8.16E-04	1.52E-08	1.30E-02	2.78E-01	LysoPC(17:0); LysoPE(0:020:0); LysoPE(20:00:0)^3^
522.3553	48.282	1.17 (1.11, 1.22)	5.77E-08	3.85 (2.37, 6.27)	1.79E-05	3.98E-03	5.78E-09	8.25E-03	2.49E-01	LysoPC(18:1(9Z)); LysoPC(18:1(11Z))^3^
494.3237	49.3839	1.15 (1.08, 1.21)	1.86E-04	2.41 (1.64, 3.54)	1.16E-03	1.04E-02	4.02E-06	9.77E-01	9.98E-01	LysoPC(16:1(9Z))^3^
149.0597	60.1071	1.17 (1.12, 1.23)	5.77E-08	3.05 (1.85, 5.03)	1.83E-03	4.75E-02	1.77E-09	5.37E-19	4.87E-16	3-Methoxy-4-hydroxyphenylacetaldehyde^2^
148.0604	89.4789	1.20 (1.10, 1.30)	2.93E-03	1.63 (1.29, 2.08)	5.88E-03	1.10E-02	8.96E-05	1.71E-01	6.41E-01	L-Glutamate^1,3^
291.0693	63.1794	0.43 (0.28, 0.66)	6.96E-03	0.91 (0.87, 0.96)	9.37E-03	2.49E-02	6.93E-09	2.01E-01	6.68E-01	Inosine^2^
205.0972	59.8331	0.92 (0.88, 0.96)	1.40E-02	0.46 (0.30, 0.71)	2.06E-02	3.15E-03	4.40E-02	7.25E-01	9.39E-01	D-Tryptophan^2,3^
244.1542	37.5565	0.82 (0.74, 0.92)	2.23E-02	0.75 (0.64, 0.89)	2.62E-02	7.84E-03	2.02E-02	7.77E-01	9.50E-01	Tiglylcarnitine^3^
620.5915	68.302	1.15 (1.07, 1.24)	9.81E-03	1.46 (1.15, 1.86)	5.05E-02	5.77E-02	1.62E-03	2.66E-01	7.40E-01	Cer(d18:122:1(13Z))^3^

The top three annotated metabolites associated with PD (log_2_FC beyond ±0.25), independently replicated in our two study populations, and unrelated to LEDD were pyroglutamic acid (log_2_FC=0.32, 95% CI=0.24, 0.41, FDR=5.6e-11), itaconate (log_2_FC=-0.33, 95% CI=-0.45, -0.22, FDR=2.2e-6), and cysteine-S-sulfate (log_2_FC=1.56, 95% CI=1.32, 1.83, FDR=8.2e-6) (Fig. 1C). Features from both columns which annotated to inosine were inversely associated with PD (C18: log_2_FC=-0.52, 95% CI=-0.78, -0.26, FDR=4.2e-3; HLIIC: log_2_FC=-1.23, 95% CI=-1.86, -0.60, FDR=6.7e-3). In the HILIC column, a series of PD associated features annotated to multiple phospholipids, including lysophosphatidylcholines (LysoPC). For example, one of the LysoPC(18:1) species, which has been implicated in PD in the past [25], was also found at higher intensity among patients in both discovery and replication in our population (log_2_FC=0.22, 95% CI=0.16, 0.29, FDR=5.8e-8), though the fold change did not pass the 0.25 threshold.

**Fig. 2:**
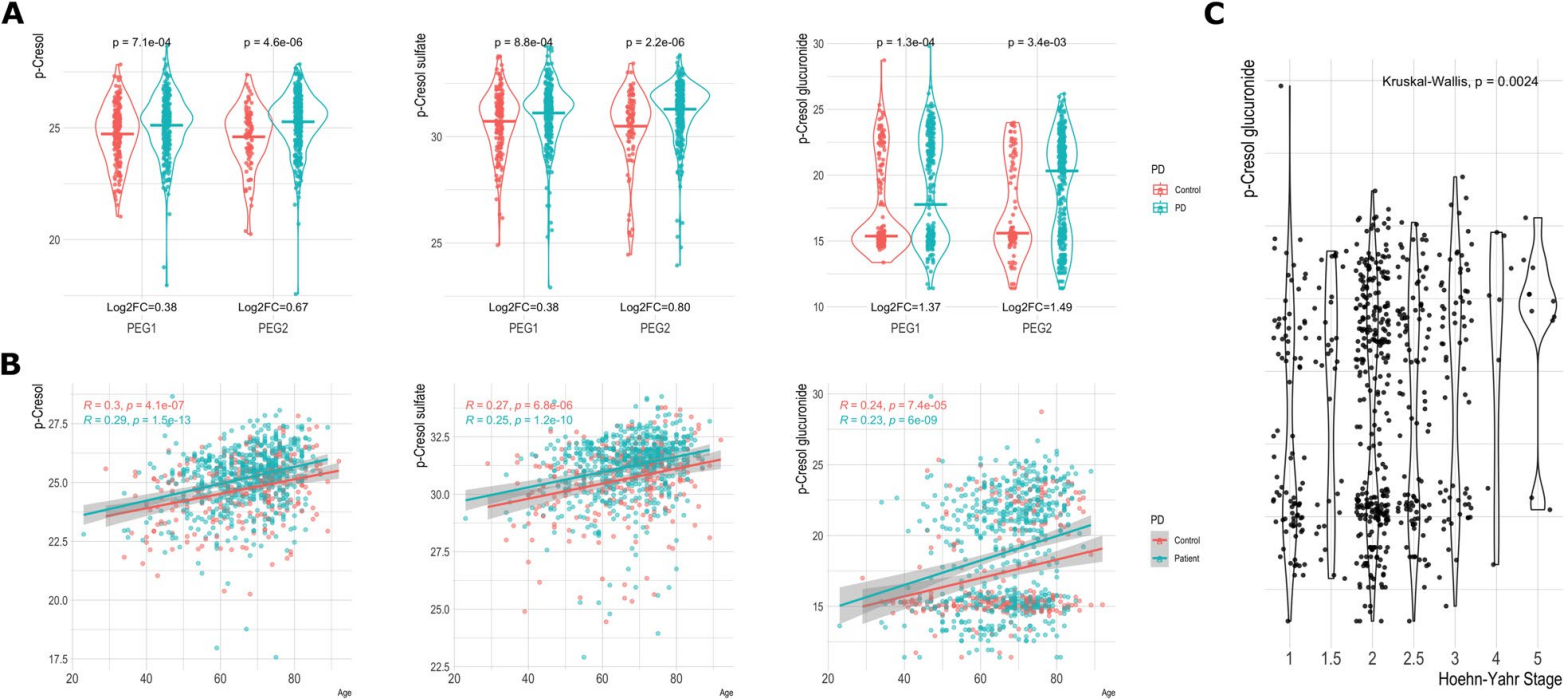
P-cresol and two p-cresol metabolites are associated with (**A**) Parkinson’s disease, (**B**) age among both PD patients and controls, and (C) Hoehn-Yahr Stage among PD patients

P-cresol and two of its metabolites, p-cresol sulfate and p-cresol glucuronide, were also found at higher intensity among the PD patients relative to controls (p-cresol log_2_FC=0.41, 95% CI=0.21, 0.60, FDR=2.8e-3). The distributions of these metabolites by PD and across discovery and replication populations are shown in Fig. 2A. The p-cresol metabolites were also correlated with age among both PD patients and controls (Fig. 2B). The PD-metabolite associations however, did not change meaningfully in the age-matched sensitivity analyses (Supplemental Table 8). Furthermore, p-cresol glucuronide was associated with a higher Hoehn Yahr (HY) stage among PD patients (beta=0.02, SE=0.007, FDR=9.5e-2; Fig. 2C).

Overall, of the PD-associated features (MWAS meta-*p*<0.05), seven were also related to HY stage among PD patients at an FDR<0.05 (115 metabolites associated at *p*<0.05), including, as expected, the PD medication metabolite, 3-O-methyldopa (Supplemental Table 9). Six PD-associated features were also associated with UPDRS-III at an FDR<0.05 (100 metabolites at *p*<0.05; Supplemental Table 10). However, other than the PD medication metabolites and p-cresol glucuronide, the features associated with either HY stage or UPDRS-III up to FDR<0.10 could not be annotated at high confidence.

### Clustering and pathway analysis

**Fig. 3:**
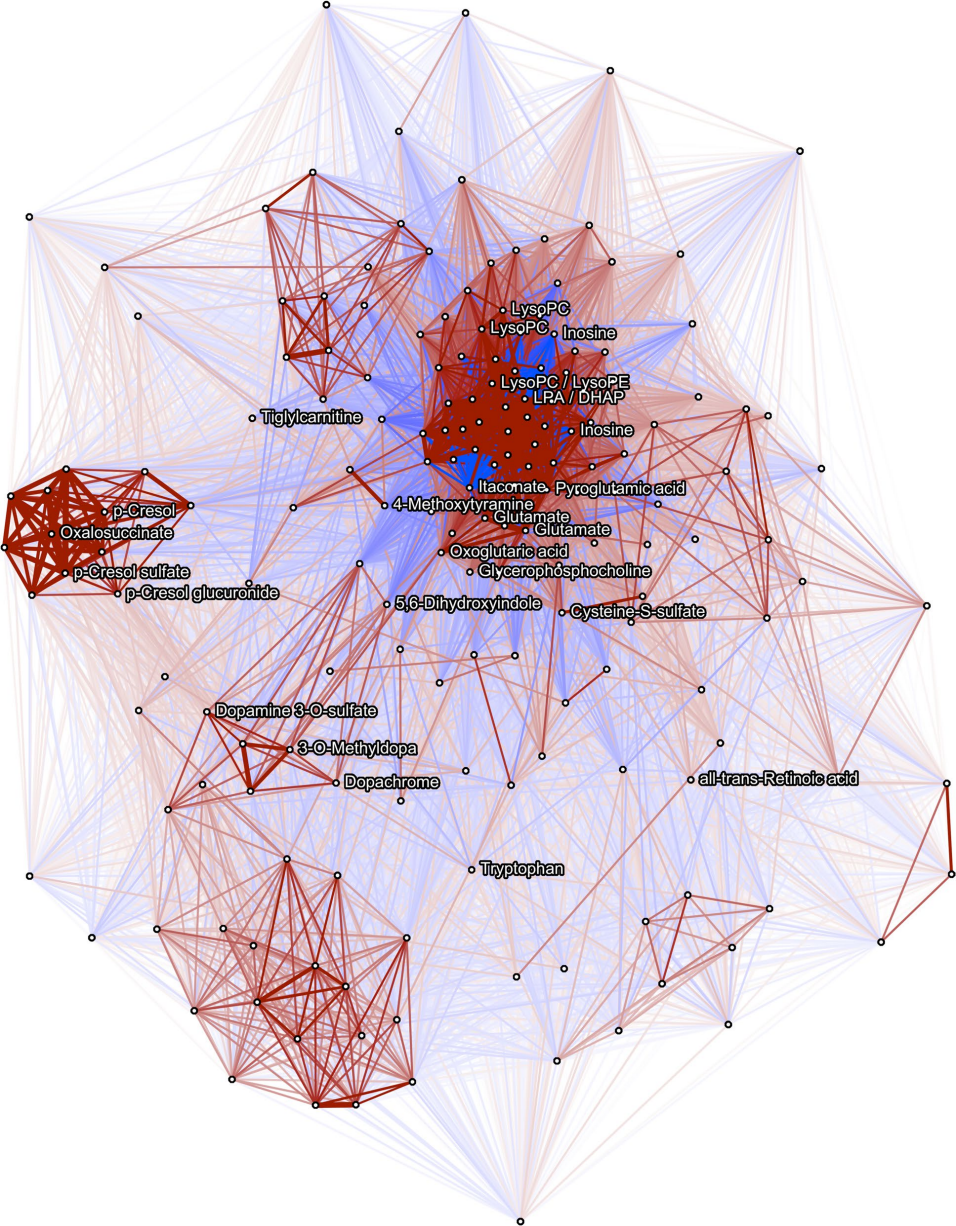
Pearson correlation network between MWAS FDR<0.05 metabolites from both the C18 and HILIC columns. |Correlations|≥0.2 shown. Features which were annotated are named to the right, while other features are shown as blank nodes

Given the interdependent nature of metabolites, we assessed correlation patterns between the PD-associated features. Figure 3 shows a Pearson-correlation based network of all FDR<0.05 MWAS features from both columns. Several highly correlated clusters of features are visible, including a PD medication related cluster, a phospholipid cluster, and a cluster of several features correlated with p-cresol.

**Fig. 4:**
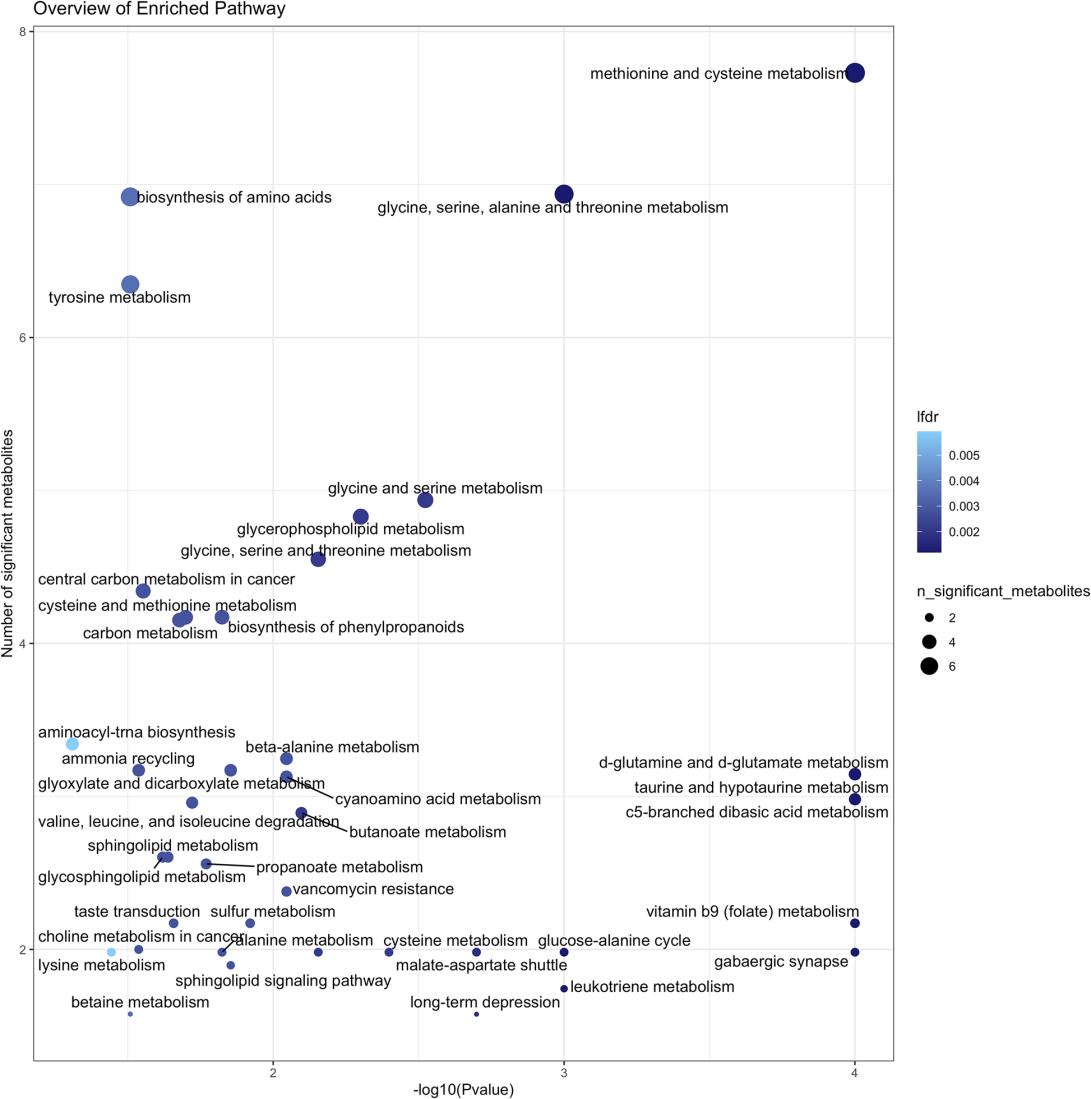
Overview of enriched pathway analysis. Based on pathway analysis of untargeted PD MWAS features using a permutation-based weighted hypergeometric test (R, *Metapone: a Bioconductor package for joint pathway testing for untargeted metabolomics data*). Pathways with *p*<0.05 are shown. Lfdr=the local FDR value for each enrichment

Multiple pathways were also enriched within the MWAS features. Based on metabolic pathway analysis, 17 pathways were significantly overrepresented at FDR<0.05 (58 at *p*<0.05; Fig. 4 and Supplemental Table 11). Glutamine and glutamate metabolism, gabaergic synapse, methionine and cysteine metabolism, glycine, serine, alanine and threonine metabolism, and leukotriene metabolism were among the most significantly overrepresented pathways. Several phospholipid pathways, including glycerophospholipid and glycosphingolipid metabolism and phospholipase-d and sphingolipid signaling pathways, were also overrepresented among the PD-associated metabolites.

### Validation of externally associated metabolites

**Fig. 5:**
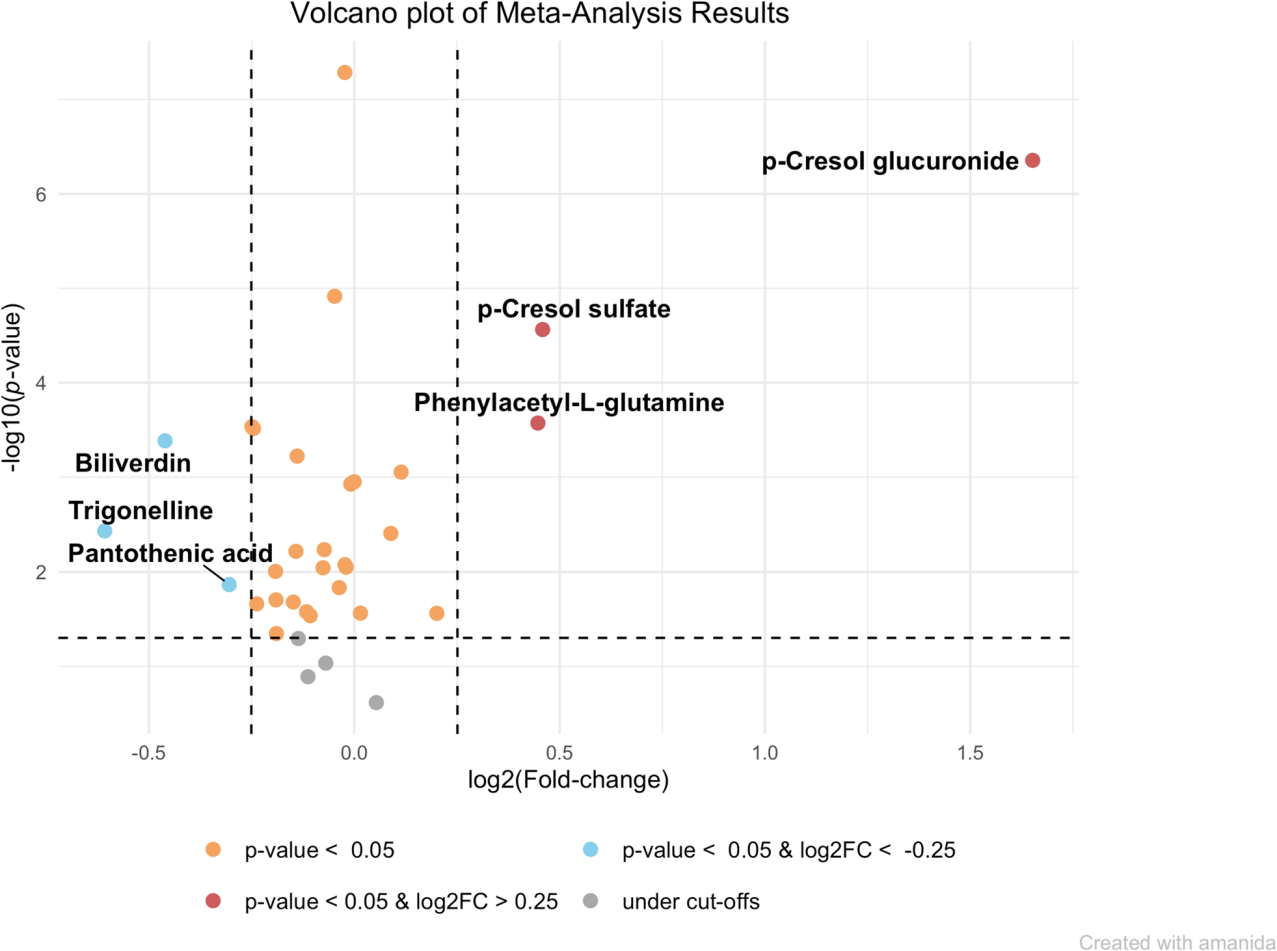
Volcano plot showing the combined fold change and *p*-values for 34 metabolites associated with PD in Shao et al, 2021 and detected in the current study LCMS data. The meta-analysis was performed using the *amanida* R package, designed to combine results when only the fold change, *p*-value, and sample size are available. *P*-values and log_2_FC are combined separately to assess validation on direction and magnitude of effect and significance separately, allowing metabolites associated in opposite directions to still be highlighted

Among the 50 metabolites distinguishing PD patients and controls found by Shao et al [7], we were able to identify 34 that were annotated to the same metabolites based on internal standards or at high confidence. Overall, 20 metabolites validated based on at least one criterion (Table 2 and Fig. 5). Six validated based on all three criteria (significance, direction of effect, and magnitude): p-Cresol glucuronide, p-Cresol sulfate, phenylacetyl-L-glutamine, trigonelline, biliverdin, and pantothenic acid. These six metabolites demonstrated the most robust associations between the two studies.

**Table 2:** External validation of metabolites previously associated with PD in an untargeted, comprehensive profiling with 226 metabolite features: Shao et al, 2021 [7] reported 50 metabolites which were differential to PD. Of these 34 were also detected and annotated at high confidence to the same metabolite in our population. Results for 20 of the 34 metabolites associated with PD are shown here, including individual study findings and combined results

**Metabolite**	**Shao et al. 2021 Table S**4		**Current Study**			***amanida* Meta-Analysis**^a^		***metaDEA* Meta-Analysis**^b^			**Validation**
	***p* value**	**Fold change**	***p* value**	**Fold change**	**95% CI**	**Combined FC**	**Adj combined *p*-value**	**Mean FC**	**SD**	**pseudo t-score**	
*p*-Cresol glucuronide	0.0021	2.15	1E-05	3.80	2.10, 6.90	3.14	7.5E-06	2.86	1.50	3.688	Direction, Significance, & Magnitude
*p*-Cresol sulfate	0.0278	1.08	8E-05	1.55	1.25, 1.93	1.37	2.3E-04	1.29	1.29	1.426	Direction, Significance, & Magnitude
FFA 20:3	0.0000	0.63	0.000	1.23	1.10, 1.36	0.98	1.8E-06	0.88	1.60	-0.381	Significance
FFA 20:4	0.0004	0.65	0.002	1.18	1.06, 1.31	0.97	1.4E-04	0.88	1.52	-0.445	Significance
Uridine	0.0300	0.91	0.003	1.18	1.06, 1.31	1.08	3.0E-03	1.04	1.20	0.274	Significance
Phenylacetyl-L-glutamine	0.0023	1.57	0.008	1.27	1.06, 1.51	1.36	1.5E-03	1.41	1.16	3.254	Direction, Significance, & Magnitude
FFA 18:2	0.0106	0.73	0.01	1.17	1.04, 1.31	1.00	3.3E-03	0.92	1.40	-0.334	Significance
Trigonelline	0.0119	0.53	0.03	0.73	0.55, 0.97	0.66	9.5E-03	0.62	1.25	-2.966	Direction, Significance, & Magnitude
Ubiquinone 1	0.0030	0.73	0.03	1.16	1.01, 1.34	0.99	3.3E-03	0.92	1.39	-0.359	Significance
FFA 14:1	0.0100	0.77	0.04	1.25	1.01, 1.53	1.06	9.5E-03	0.98	1.41	-0.079	Significance
Kynurenine	0.0113	0.86	0.05	0.93	0.86, 1.00	0.91	1.3E-02	0.89	1.06	-2.855	Direction & Significance
Biliverdin	0.0003	0.7	0.08	0.74	0.52, 1.04	0.73	1.8E-03	0.72	1.04	-11.837	Direction, Significance, & Magnitude
Pantothenic acid	0.0174	0.85	0.09	0.79	0.61, 1.03	0.81	2.2E-02	0.82	1.05	-5.440	Direction, Significance, & Magnitude
FFA 20:2	0.0090	0.76	0.09	1.12	0.98, 1.28	0.98	1.6E-02	0.92	1.32	-0.415	Significance
Indolelactic acid	0.0109	0.81	0.09	0.91	0.82, 1.02	0.88	1.7E-02	0.86	1.09	-2.620	Direction & Significance
FFA 22:5	0.0002	0.63	0.17	1.09	0.97, 1.22	0.91	2.3E-03	0.83	1.47	-0.686	Significance
FFA 19:1	0.0179	0.78	0.18	1.15	0.94, 1.40	1.01	3.3E-02	0.95	1.32	-0.280	Significance
Cortisol	0.0165	1.21	0.19	1.12	0.94, 1.33	1.15	3.3E-02	1.16	1.06	3.932	Direction & Significance
FFA 20:0	0.0307	0.87	0.19	0.88	0.72, 1.07	0.88	5.1E-02	0.87	1.01	-23.371	Direction & Significance
cis-Aconitic acid	0.0078	0.83	0.25	0.90	0.75, 1.08	0.88	2.9E-02	0.86	1.06	-3.602	Direction & Significance

^a^*Amanida*: meta-analysis done using *amanida* R package, designed to combine results when only the fold change, *p*-value, and sample size are available. For significance evaluation using the statistic result *p*-value, *amanida* uses a weighted *p*-values combination, which is a variant of Fisher’s method. “A gamma distribution is used to assign non-integral weights proportional to study size to each *p*-value. The logarithmically transformed fold-change values are averaged with weighting by study size.” *Amanida* reference [23]

^b^*metaDE*: meta-analysis done using *metaDEA* R package, which averages the study-specific log2FCs and calculates the SD. The "pseudo t-score" is the ratio of mean log2FC to the SD of the log2FCs divided by the square root of the number of comparisons. “This statistic is negative for metabolites with lower abundance and positive for higher abundance and its absolute value will be higher for those metabolites with high and consistent changes across comparisons, and lower for inconsistent and variable fold changes (e.g., upregulated in some datasets and downregulated in others) that are found only in a few comparisons.” *metaDEA* reference [24]

Significance: individual study *p*<0.25 and combined *p*-value<0.05

Direction: individual study fold changes are at least ±5% and directions match between the two studies

Magnitude: Combined log_2_FC > 0.25 or < -0.25

Five metabolites were validated based on direction and significance, but the log_2_FC did not reach ±0.25. This includes FFA 20:0, which had the highest ranked validation based on the *metaDEA* pseudo t-score, meaning the fold changes were most similar between the two studies (FC=0.87 and FC=0.88, see Table 2). Nine metabolites, mostly free fatty acids, only agreed on significance but the studies reported opposite directions of effect: FFA 20:3, FFA 20:4, uridine, FFA 18:2, ubiquinone 1, FFA 14:1, FFA 20:2, FFA 22:5, FFA 19:1.

Results for all 34 metabolites are shown in Supplemental Table 12.

## Discussion

Using high-resolution, untargeted serum metabolic profiling based on LC-HRMS, we identified 296 metabolite features and 17 metabolic pathways associated with PD, 134 of which had a log_2_FC greater than ±0.5. Importantly, we also assessed external validation of 34 metabolites previously associated with PD in an untargeted scan of 226 metabolites [7]. We combined our results and the Shao et al. data, the only other large-scale (n>400) untargeted screen of blood-based metabolite features from LCMS. In total, between the two studies, 20 metabolites were highlighted, with six showing the most robust evidence for association when considering significance, direction, and magnitude of effect (p-Cresol glucuronide, p-Cresol sulfate, phenylacetyl-L-glutamine, trigonelline, biliverdin, and pantothenic acid).

Our untargeted metabolomics approach broadly implicated amino acid metabolism and phospholipid pathways as important in PD, along with multiple individual metabolites with compelling links to neurodegeneration. Serum was collected from PD patients early in disease and compared to community controls. These metabolites and pathways therefore may reflect disturbances due to disease pathogenesis and progression as well as compensatory or reactive mechanisms caused by disease or treatment.

One of the strengths of our study is that we assessed metabolite profiles from PD patients recruited early in disease who were undergoing a range of treatment courses. Thus, we were able to assess the relationship between all metabolite features and levodopa equivalent daily dose to determine which features associated with PD were also associated with medication use. Predictably, PD patients differed from controls most strongly in terms of levodopa medication metabolites or metabolites involved in dopamine metabolism. Network analysis further showed the PD medication metabolites clustered together, as expected, but were not significantly correlated with other PD-related metabolites. Several other pathways and specific metabolites were implicated independent of levodopa medication use. Another noteworthy metabolite that attests to the validity of our analyses is inosine, which is a precursor to urate, with anti-inflammatory properties. We observed inosine at lower intensity among the patients relative to controls. This is in line with previous studies and the notion that lower uric acid level may be involved in faster PD progression, which has led to inosine supplementation trials [26–29]. Though these trials have not shown success.

One of the more intriguing findings was p-cresol and its two metabolites, p-cresol sulfate and p-cresol glucuronide. We observed higher intensities of the metabolites among PD patients relative to controls in both discovery and replication populations. Furthermore, this was external validation for p-cresol sulfate and p-cresol glucuronide, which were also positively associated with PD by Shao et al [7]. In fact, p-cresol glucuronide showed the strongest association when combining results across the two studies, with over three-fold difference between patients and controls. Moreover, the intensity of all three p-cresol metabolites were positively related to age. P-cresol glucuronide was also related to higher motor symptom scores.

P-cresol is an exogenous uremic toxin primarily produced by gut bacteria, which express p-cresol synthesizing enzymes that are not produced by human cells. It has been shown to induce oxidative stress and inflammation in vitro [30]. Interestingly, two smaller studies previously found higher levels of p-cresol and p-cresol sulfate in the cerebrospinal fluid of PD patients [7, 31, 32]. Additionally, multiple studies have also linked p-cresol with autism [33–35] and altered brain dopamine metabolism in neurodevelopment [36]. Furthermore, gut dysbiosis has been linked to both PD and autism, including among our own patients [37], with some research even indicating that misfolded a-synuclein retrogradely propagates from the enteric to the central nervous system [38]. The positive age association with older age we detected in this study has been reported previously [39]. Interestingly in the same study, p-cresol sulfate levels were not correlated with measured levels of its pre-cursor tyrosine. P-cresol and its’ metabolites therefore represent compelling targets for future mechanistic research.

Several tricarboxylic acid (TCA) cycle metabolites were also implicated as relevant to PD in our MWAS, confirming several previous targeted metabolomics studies [40]. PD patients had higher relative abundance of oxoglutaric acid (e.g. alpha-ketoglutarate) and lower levels of itaconate. Pantothenic acid was inversely associated with PD in both our study and Shao et al, and among the six features that externally validated based on all three criteria. Pantothenic acid is necessary to synthesize coenzyme A (CoA), which is involved in the TCA cycle with alpha-ketoglutarate. Lower levels of pantothenic acid have been found in several regions of PD brains relative to controls, including the cerebellum and substantia nigra [41]. cis-Aconitic acid, another TCA metabolite, was also confirmed in our external validation and meta-analysis.

In terms of itaconate, aside from implications for energy metabolism, highly pertinent as PD involves mitochondrial dysfunction, the metabolite holds key roles in immunometabolism (e.g., changes of metabolic pathways within immune cells) [42, 43]. Itaconate is a mitochondrial metabolite, produced in high amounts by macrophages and monocytes by diverting aconitate away from the TCA cycle during inflammatory activation [43]. The primary function appears to be anti-inflammatory, supported by human studies showing that low levels of plasma itaconate coincide with excessive inflammation [43]. Inflammation and neuroinflammation are principal features of PD. Thus, it is quite interesting that in both of our discovery and replication populations we found lower relative levels of this important anti-inflammatory immunometabolite among the PD patients.

We further identified several amino acids as differentially abundant in PD. Glutamate and several connected metabolites, including phenylacetyl-L-glutamine (e.g., phenylacetylglutamine) and pyroglutamic acid (PGA), had a higher relative abundance in patients’ serum relative to controls, with the glutamine and glutamate metabolism pathways significantly overrepresented. Phenylacetylglutamine was one of the top metabolites from the external validation. Furthermore, this metabolite was also linked to PD in a smaller metabolomics study [8]. Phenylacetylglutamine is a gut-microbially derived metabolite formed from protein putrefaction of phenylalanine and tyrosine by the gut microbiota [44], again implicating the gut microbiome in PD.

Pyroglutamic acid (PGA) is an endogenous metabolite derived from glutamate and linked to glutathione turnover [45, 46]. Elevated serum PGA therefore may be related to perturbed glutathione metabolism. Low levels of the antioxidant glutathione are an early neuronal biochemical finding in PD [47]. But increased systemic levels of PGA may reflect an upregulation of glutathione metabolism to counter inflammatory states and oxidative stress in PD. Furthermore, the neurotransmitter glutamate itself has been linked to PD pathogenesis, with several, though not all, studies reporting increased blood-measured levels of glutamate [48, 49].

Other amino acid metabolic pathways, including methionine and cysteine metabolism, glycine, serine, alanine and threonine metabolism, and valine, leucine, and isoleucine degradation were also overrepresented among PD-associated features, with individual metabolites like serine, isoleucine, and tryptophan observed in higher relative abundance among the PD patients. Kynurenine, a metabolite of tryptophan, was one of the top metabolites from our combination of results with Shao et al data. It has diverse functions related to immune activation and regulation [50, 51]. Two smaller metabolomic studies have also linked kynurenine to PD [52, 53]. The patients also had higher levels of cysteine-S-sulfate, a purportedly brain damaging metabolite involved in sulfite oxidase deficiency [54]. Branched chain amino acids (BCAAs), including leucine, isoleucine, and valine, have also been linked to PD, as BCAAs are involved in energy metabolism, preventing oxidative damage, and regulation of protein synthesis [5].

Lipid pathways and metabolites were also implicated with PD by our MWAS. Glycerophospholipid along with glycosphingolipid and sphingolipid metabolism were enriched in pathway analyses. Metabolites including glycerophosphocholine, several lysophosphotidylcholines (LysoPC), and a ceramide, were all observed at higher intensities among the patients relative to controls. The lipid profile in PD has received a great deal of interest in recent years, in part due the identification of *GBA* variants in PD GWAS. The glucosylceramidase-beta (*GBA*) gene, which encodes the lysosomal enzyme glucocerebrosidase (GCase), has directly connected lipid and sphingolipid metabolism to PD pathogenesis [55]. PD pathogenic mechanisms linked to lipid metabolism include oxidative stress, inflammation and immune system signaling, pro-apoptotic processes, and interaction with a-synuclein biology, among others. Furthermore, alterations in serum, plasma, and brain measured phospholipids and sphingolipids have been widely reported in PD [56]. For instance, LysoPC(18:1), implicated in our MWAS, has also been found at higher levels in the substantia nigra in animal models of PD [25]. Interestingly, Cer(d18) metabolites, one of which was observed at higher intensity among our PD patients, have also been associated with physical frailty among older adults [57].

Several free fatty acids (FFA) were also highlighted in our combination with Shao et al., with one species (FFA 20:0) showing an inverse association in both studies. However, several were confirmed based only on significance, as associations were in opposite directions. This is possibly due to L-dopa use. L-dopa has previously been reported to increase plasma FFAs, while patients with low serum levels of L-dopa did not show a significant increase in plasma FFAs [58]. Shao et al compared untreated, drug-naïve patients to controls, while our patients were taking varying levels of L-dopa.

Trigonelline and biliverdin were also among the externally validated metabolites. Patients from both populations had lower levels of trigonelline, which has shown neuroprotective action against PD along with other neurologic diseases including Alzheimer's, stroke, and depression [59]. Biliverdin is a breakdown product of the pro-oxidant heme, which is further oxidized to bilirubin. It has been linked to PD in other studies as well, implicating oxidative stress and bile acid pathways [60, 61]. Cortisol was also implicated in the external validation, with patients from both studies showing higher levels. Cortisol and stress pathways have also been described in PD [62].

Overall, our study is among the largest untargeted high-resolution metabolomics study of PD to date with metabolic profiles from independent discovery and replication case-control study populations allowing for validation of associated features. However, a notable limitation of the untargeted LC-HRMS technology is feature annotation. LC-HRMS provides metabolite features, many of which are not identified and can only be annotated based on m/z and retention time parameters from large databases (e.g. HMDB) and with consideration of feature correlation structures. While this does allow high confidence annotation, future research will be needed to identify features with certainty. For instance, para-, ortho-, and meta-cresol are all isomers. We have labeled the cresol metabolite as p-cresol due to co-occurrence with the p-cresol metabolites, p-cresol sulfate and p-cresol glucuronide, and because of the three exogenous isomers, it is produced in humans via gut microbes. However, future studies will be needed to resolve the isomers. Furthermore, one-to-many matching and no matching add further uncertainty to feature annotation. Many of the features associated with PD in our MWAS, including some of the most significantly associated features, could not yet be annotated. As reference libraries grow, including HMDB and KEGG, and experiments continue, hopefully in the future these metabolites will be annotated. Additionally, the metabolome measurements were also based on a single blood-draw. Future longitudinal studies will be very informative in disentangling which if any metabolites implicated here are causally related to PD versus disease progression or reactive mechanisms. Still, our study was able to externally validate the associations for several metabolites previously reported in a different population, (race/ethnicity, diet, medication status, a clinical-based recruitment). Such validation, from separate, agnostic investigations in populations with different diets, medication status (drug-naïve versus L-dopa use), countries and lifestyles supports a robust association of the features with PD [17]. The eleven metabolites identified in both large-scale metabolomics studies, with the same direction of effect, represent compelling targets for further investigation.

In conclusion, based on this untargeted high-resolution, serum metabolic profiling from LC-HRMS, we have implicated over 200 individual metabolite features in PD along with multiple metabolic pathways. Several metabolite hits associated pathways known to be disrupted in PD, including amino acid and lipid metabolism. We present many novel findings, including for itaconate, connecting impaired anti-inflammatory signaling through immunometabolism, while providing external confirmation for multiple other metabolites and association with PD, including the three p-cresol metabolites and phenylacetyl-L-glutamine, linking gut microbial activity to PD.

### Supplementary Information

**Additional file 1: Supplementary Tables.**

**Additional file 2:** **Supplemental Figure 1.** C18 negative column metabolomics processing: Sum of metabolite intensities across samples colored by batch & sample type, before pre-processing (log transformation, quantile normalization, ComBat batch correction). LCMS was run across 30 batches (*n*=46); machine was reset after 694 samples (i.e., samples ran in two larger groups of *n*=694 samples, each with 15 smaller batches within run). Run, batch, and drift effects are apparent in raw data.

**Additional file 3:** **Supplemental Figure 2.** C18 negative column after metabolomics processing. Raw c18 data was log transformation, quantile normalized, followed by ComBat for batch correction. LCMS was run across 30 batches (*n*=46); machine was reset after 694 samples (i.e., samples ran in two larger groups of *n*=694 samples, each with 15 smaller batches within run. While there are several apparent outliers, after processing, technical variation has been removed.

**Additional file 4:** **Supplemental Figure 3.** C18 negative column metabolomics processing: Principal component analysis of raw and processed metabolomics data. PC variation primarily explained by LCMS run in raw data. After correction, sample type (quality control sample versus the study serum samples) primarily explains variation.

**Additional file 5:** **Supplemental Figure 4.** HILIC positive column metabolomics processing: Sum of metabolite intensities across samples colored by batch & sample type before and after pre-processing (log transformation, quantile normalization, ComBat batch correction). LCMS ran in across 30 batches (*n*=46); machine was reset after 694 samples (i.e., samples ran in two larger groups of *n*=694 samples, each with 15 smaller batches within run). Run, batch, and drift effects are apparent in raw data. While there are several apparent outliers, after processing, the technical variation has been removed.

**Additional file 6:** **Supplemental Figure 5.** HILIC positive column metabolomics processing: Principal component analysis of metabolomics data after median normalization and ComBat correction for batch effects. PC variation primarily explained by batch in raw data, after correction sample type (quality control sample versus the population-based serum samples) primarily explains variation. However, there are two apparent clusters of population-based serum samples, potentially explained by non-biologic (PD) technical variation (see Supplemental Fig. 6).

**Additional file 7:** **Supplemental Figure 6.** HILIC positive PCA of processed data, colored by different covariates. No distinguishing variables to describe the different clusters of study samples, though there is some separation by year of sample. Note gray indicates the QC samples. Therefore, we additionally corrected for inclusion in this cluster, as variation appears technical and is very influential in MWAS (Supplemental Fig. 7).

**Additional file 8:** **Supplemental Figure 7.** HILIC positive metabolomics data after processing: Log transformation, quantile normalization, ComBat batch correction, and additional adjustment for unexplained PC.

**Additional file 9:** **Supplemental Figure 8.** Comparison of MWAS results (log2FC) when pooling the data and the processing (e.g., normalization and combat batch correction) versus processing and analyzing the data independently. (A & B) HILIC and C18 features: comparing the pooled processing logFC to a meta-analysis combining the results from each run, which was processed independently. (C & D) HILIC and C18 features: comparing the stratified results from run1 and run2, with each run was processed independently.

**Additional file 10:** **Supplemental Figure 9.** Volcano plots for the HILIC and C18 analysis with each run was processed (e.g., normalization and combat batch correction) and analyzed independently.

**Additional file 11:** **Supplemental Figure 10.** Top metabolite results shown by HRMS run. Processing / normalization on each run independently. Mean comparisons of the crude data, shown on the log2 scale, and compared with a Wilcoxon test. Supplemental Tables 2 and 3 show results from adjusted models.

**Additional file 12:** **Supplemental Figure 11.** Top metabolite results shown by HRMS run. Processing / normalization on pooled data. Mean comparisons of the crude data, shown on the log2 scale, and compared with a Wilcoxon test. Supplemental Tables 2 and 3 show results from adjusted models.

## Data Availability

The metabolomics data used in this study are available on metabolomics workbench under the project title "Untargeted serum metabolomics in the Parkinson's Environment and Genes (PEG) Study".
