# Supplementary material for: Untargeted serum metabolomics reveals novel metabolite associations and disruptions in amino acid and lipid metabolism in Parkinson’s disease
Source: Mol Neurodegener. 2023 Dec 19;18:100. doi: 10.1186/s13024-023-00694-5 (PMC10731845; doi:10.1186/s13024-023-00694-5)
Supplement: Supplementary file 2 — Additional file 2: Supplemental Figure 1. C18 negative column metabolomics processing: Sum of metabolite intensities across samples colored by batch & sample type, before pre-processing (log transformation, quantile normalization, ComBat batch correction). LCMS was run across 30 batches (n=46); machine was reset after 694 samples (i.e., samples ran in two larger groups of n=694 samples, each with 15 smaller batches within run). Run, batch, and drift effects are apparent in raw data. [file 13024_2023_694_MOESM2_ESM.docx]

**
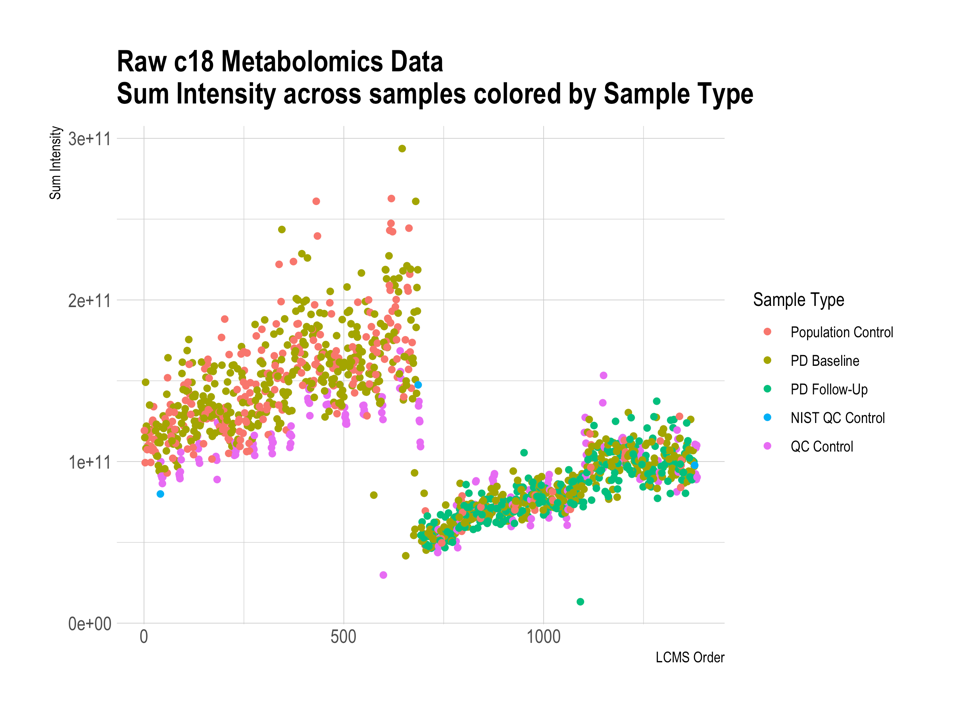
Supplemental Figures**

**
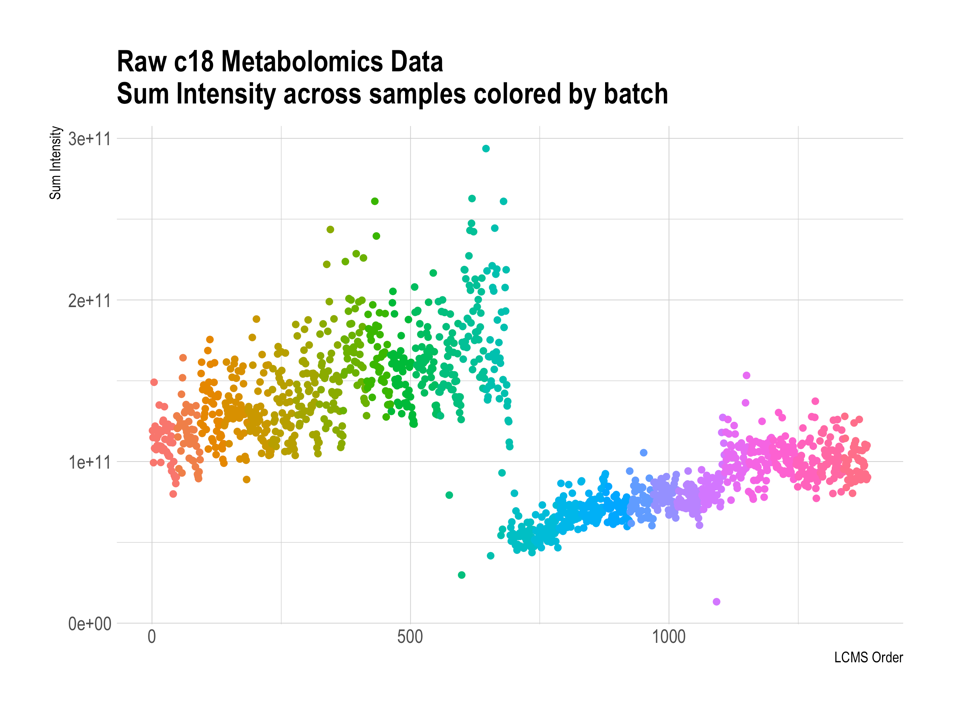
**

**Supplemental Figure 1.** **C18 negative column metabolomics processing:** Sum of metabolite intensities across samples colored by batch & sample type, before pre-processing (log transformation, quantile normalization, ComBat batch correction). LCMS was run across 30 batches (n=46); machine was reset after 694 samples (i.e., samples ran in two larger groups of n=694 samples, each with 15 smaller batches within run). Run, batch, and drift effects are apparent in raw data.
