# Supplementary material for: Untargeted serum metabolomics reveals novel metabolite associations and disruptions in amino acid and lipid metabolism in Parkinson’s disease
Source: Mol Neurodegener. 2023 Dec 19;18:100. doi: 10.1186/s13024-023-00694-5 (PMC10731845; doi:10.1186/s13024-023-00694-5)
Supplement: Supplementary file 3 — Additional file 3: Supplemental Figure 2. C18 negative column after metabolomics processing. Raw c18 data was log transformation, quantile normalized, followed by ComBat for batch correction. LCMS was run across 30 batches (n=46); machine was reset after 694 samples (i.e., samples ran in two larger groups of n=694 samples, each with 15 smaller batches within run. While there are several apparent outliers, after processing, technical variation has been removed. [file 13024_2023_694_MOESM3_ESM.docx]

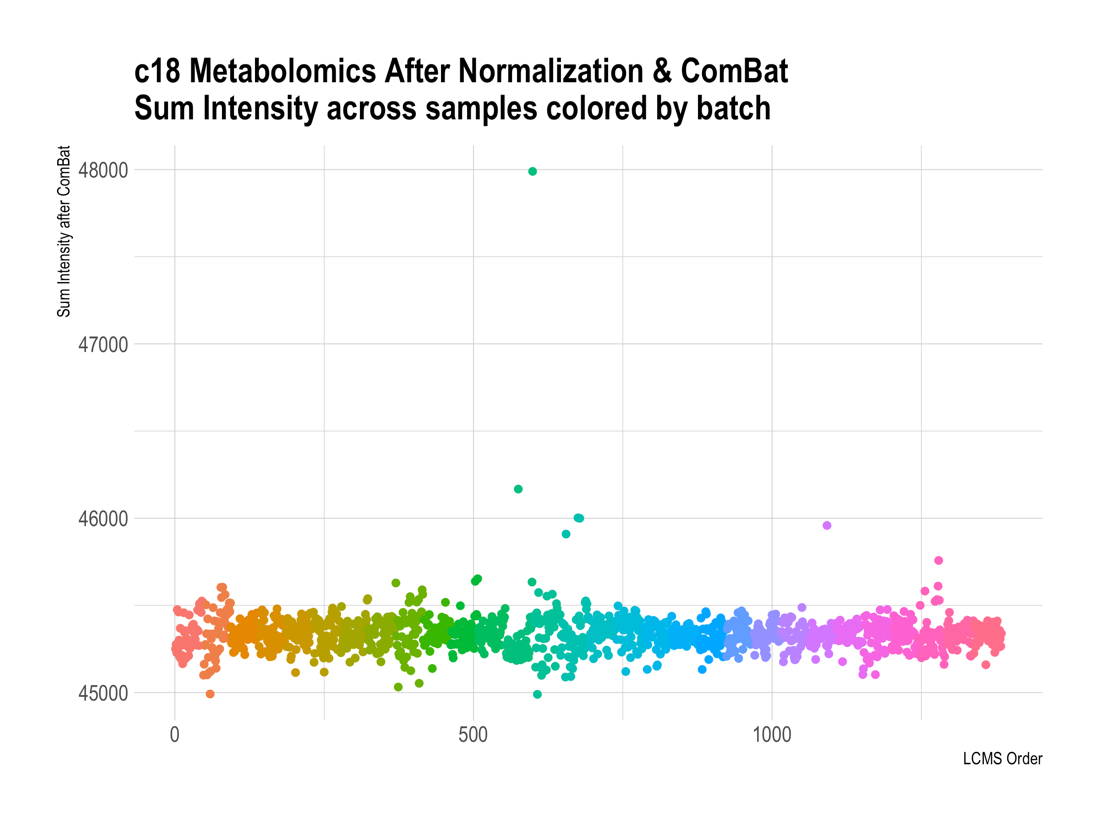
**
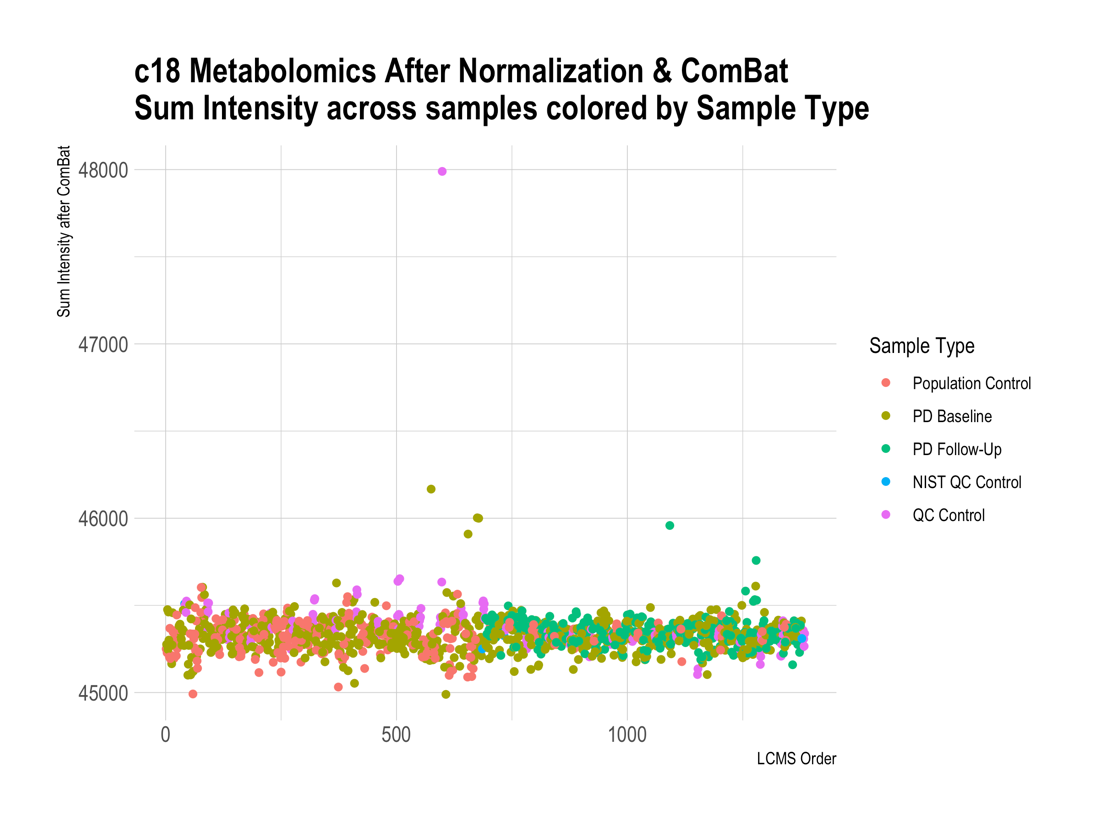
**

**Supplemental Figure 2.** **C18 negative column after metabolomics processing.** Raw c18 data was log transformation, quantile normalized, followed by ComBat for batch correction. LCMS was run across 30 batches (n=46); machine was reset after 694 samples (i.e., samples ran in two larger groups of n=694 samples, each with 15 smaller batches within run. While there are several apparent outliers, after processing, technical variation has been removed.
