# Supplementary material for: Untargeted serum metabolomics reveals novel metabolite associations and disruptions in amino acid and lipid metabolism in Parkinson’s disease
Source: Mol Neurodegener. 2023 Dec 19;18:100. doi: 10.1186/s13024-023-00694-5 (PMC10731845; doi:10.1186/s13024-023-00694-5)
Supplement: Supplementary file 4 — Additional file 4: Supplemental Figure 3. C18 negative column metabolomics processing: Principal component analysis of raw and processed metabolomics data. PC variation primarily explained by LCMS run in raw data. After correction, sample type (quality control sample versus the study serum samples) primarily explains variation. [file 13024_2023_694_MOESM4_ESM.docx]

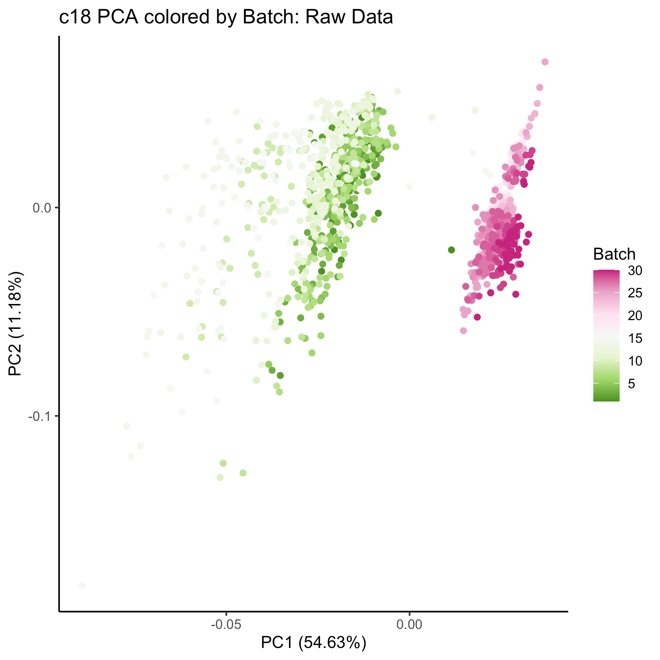

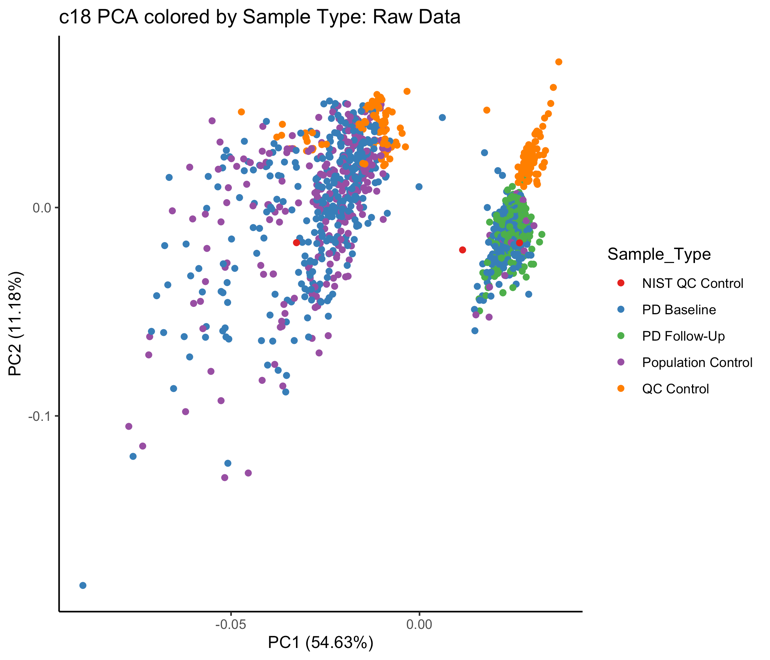


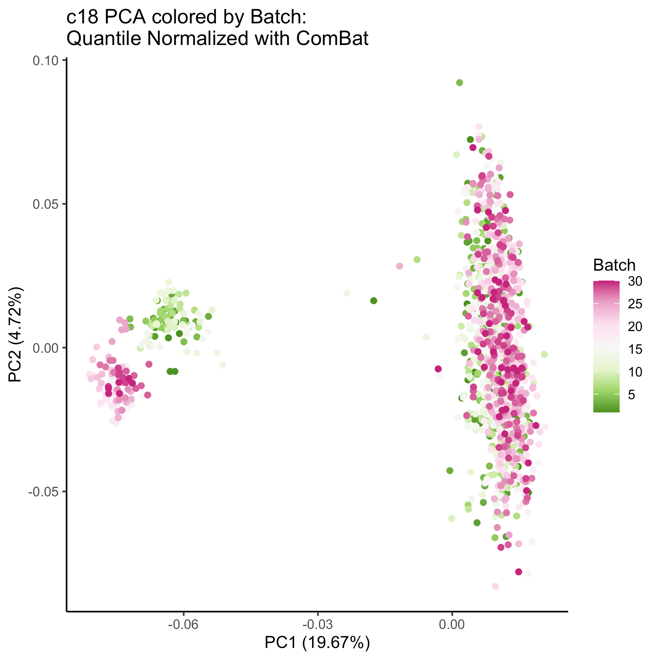

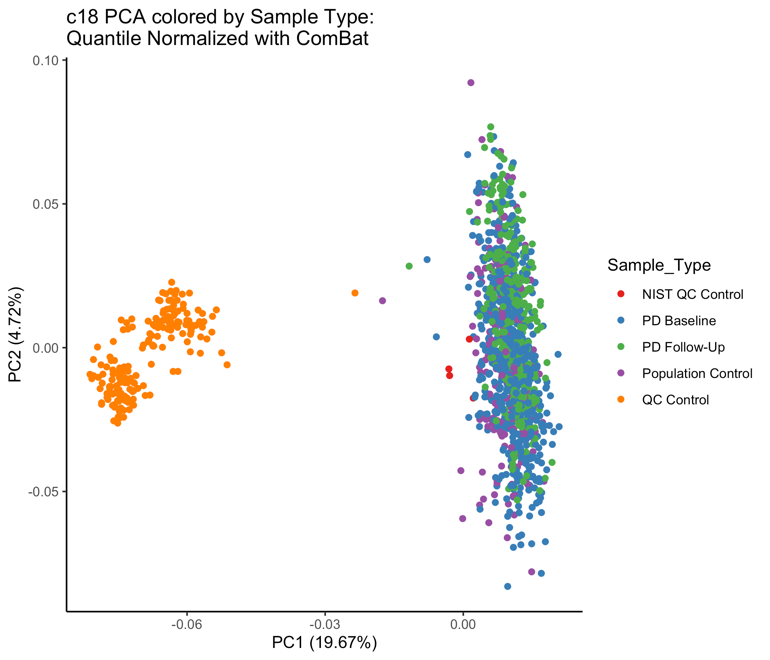


**Supplemental Figure 3. C18 negative column metabolomics processing:** Principal component analysis of raw and processed metabolomics data. PC variation primarily explained by LCMS run in raw data. After correction, sample type (quality control sample versus the study serum samples) primarily explains variation.
