# Supplementary material for: Untargeted serum metabolomics reveals novel metabolite associations and disruptions in amino acid and lipid metabolism in Parkinson’s disease
Source: Mol Neurodegener. 2023 Dec 19;18:100. doi: 10.1186/s13024-023-00694-5 (PMC10731845; doi:10.1186/s13024-023-00694-5)

**Supplemental Figure 4.** **HILIC positive column metabolomics processing:** Sum of metabolite intensities across samples colored by batch & sample type before and after pre-processing (log transformation, quantile normalization, ComBat batch correction). LCMS ran in across 30 batches (n=46); machine was reset after 694 samples (i.e., samples ran in two larger groups of n=694 samples, each with 15 smaller batches within run). Run, batch, and drift effects are apparent in raw data. While there are several apparent outliers, after processing, the technical variation has been removed.


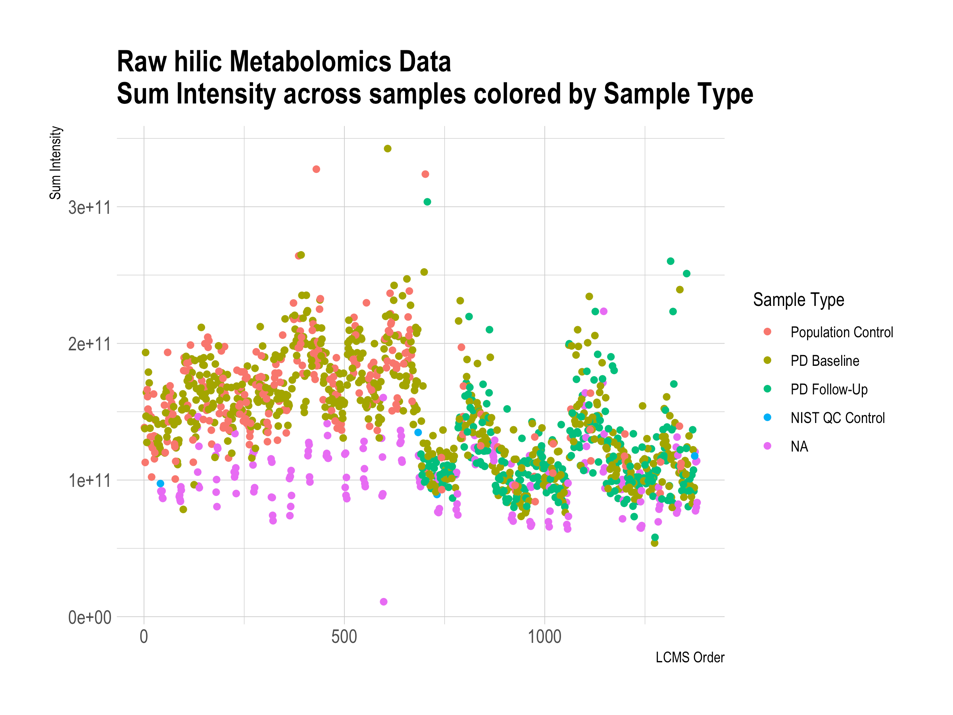

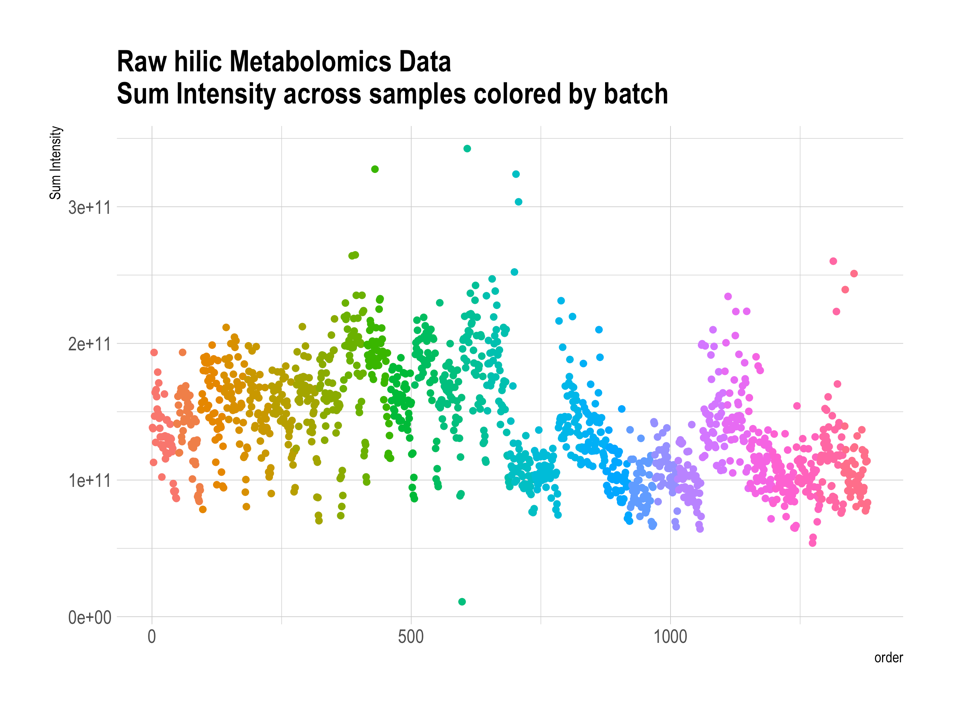

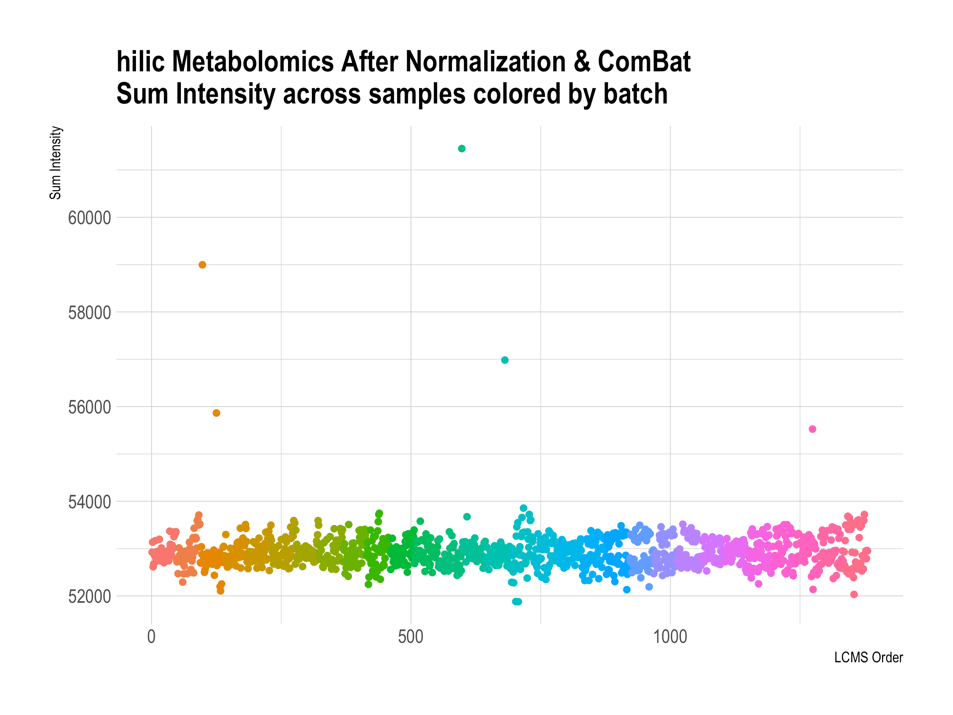

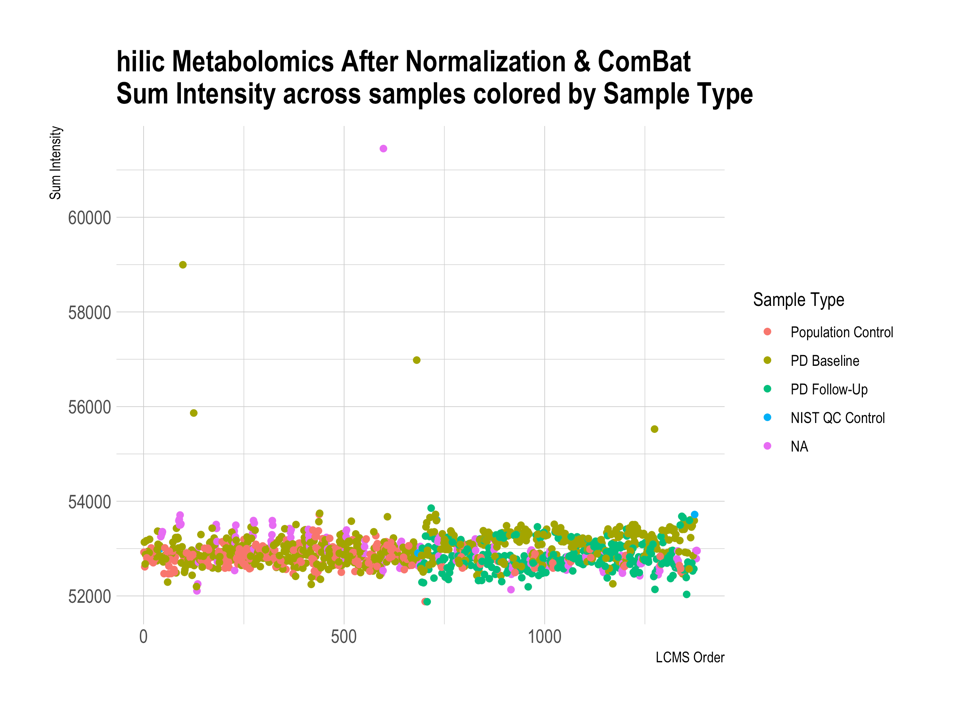

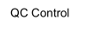

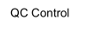

Supplement: Supplementary file 5 — Additional file 5: Supplemental Figure 4. HILIC positive column metabolomics processing: Sum of metabolite intensities across samples colored by batch & sample type before and after pre-processing (log transformation, quantile normalization, ComBat batch correction). LCMS ran in across 30 batches (n=46); machine was reset after 694 samples (i.e., samples ran in two larger groups of n=694 samples, each with 15 smaller batches within run). Run, batch, and drift effects are apparent in raw data. While there are several apparent outliers, after processing, the technical variation has been removed. [file 13024_2023_694_MOESM5_ESM.docx]
