# Supplementary material for: Untargeted serum metabolomics reveals novel metabolite associations and disruptions in amino acid and lipid metabolism in Parkinson’s disease
Source: Mol Neurodegener. 2023 Dec 19;18:100. doi: 10.1186/s13024-023-00694-5 (PMC10731845; doi:10.1186/s13024-023-00694-5)
Supplement: Supplementary file 6 — Additional file 6: Supplemental Figure 5. HILIC positive column metabolomics processing: Principal component analysis of metabolomics data after median normalization and ComBat correction for batch effects. PC variation primarily explained by batch in raw data, after correction sample type (quality control sample versus the population-based serum samples) primarily explains variation. However, there are two apparent clusters of population-based serum samples, potentially explained by non-biologic (PD) technical variation (see Supplemental Fig. 6). [file 13024_2023_694_MOESM6_ESM.docx]

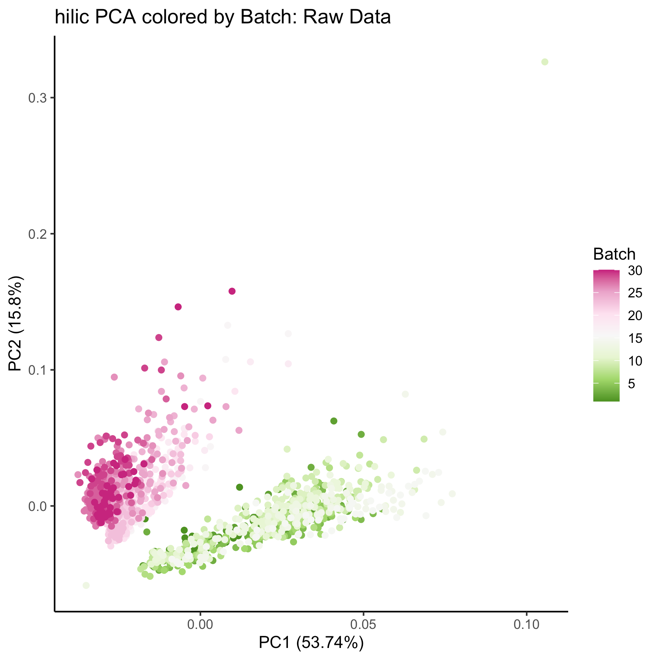

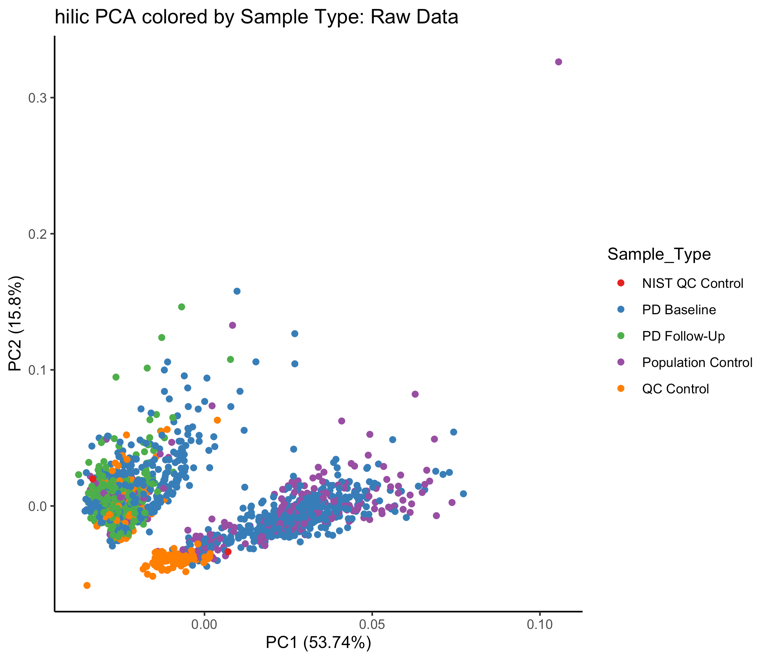


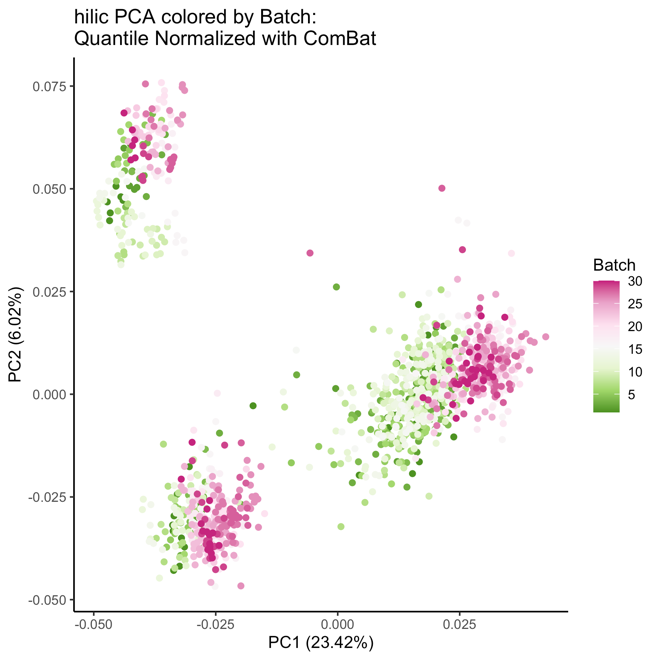

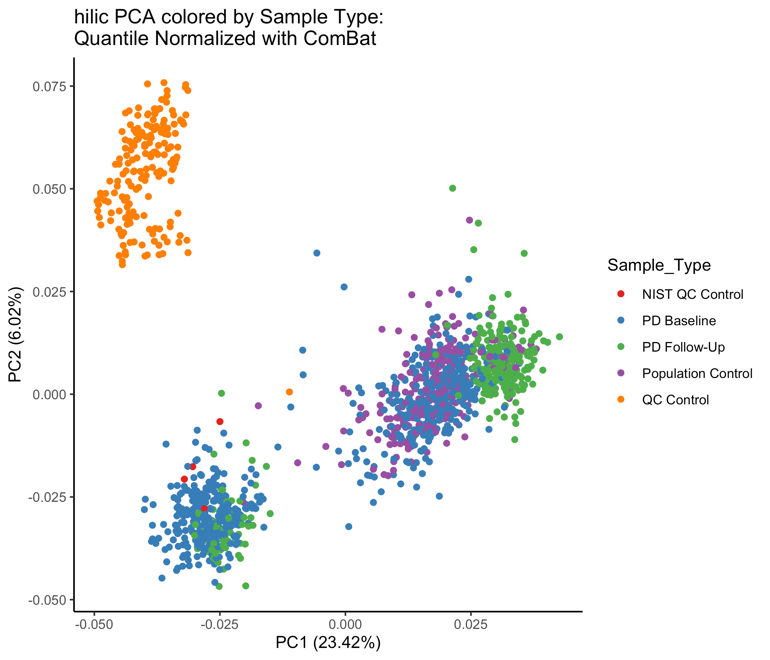


**Supplemental Figure 5. HILIC positive column metabolomics processing:** Principal component analysis of metabolomics data after median normalization and ComBat correction for batch effects. PC variation primarily explained by batch in raw data, after correction sample type (quality control sample versus the population-based serum samples) primarily explains variation. However, there are two apparent clusters of population-based serum samples, potentially explained by non-biologic (PD) technical variation (see **Supplemental Figure 6**).
