# Supplementary material for: Untargeted serum metabolomics reveals novel metabolite associations and disruptions in amino acid and lipid metabolism in Parkinson’s disease
Source: Mol Neurodegener. 2023 Dec 19;18:100. doi: 10.1186/s13024-023-00694-5 (PMC10731845; doi:10.1186/s13024-023-00694-5)
Supplement: Supplementary file 7 — Additional file 7: Supplemental Figure 6. HILIC positive PCA of processed data, colored by different covariates. No distinguishing variables to describe the different clusters of study samples, though there is some separation by year of sample. Note gray indicates the QC samples. Therefore, we additionally corrected for inclusion in this cluster, as variation appears technical and is very influential in MWAS (Supplemental Fig. 7). [file 13024_2023_694_MOESM7_ESM.docx]

**
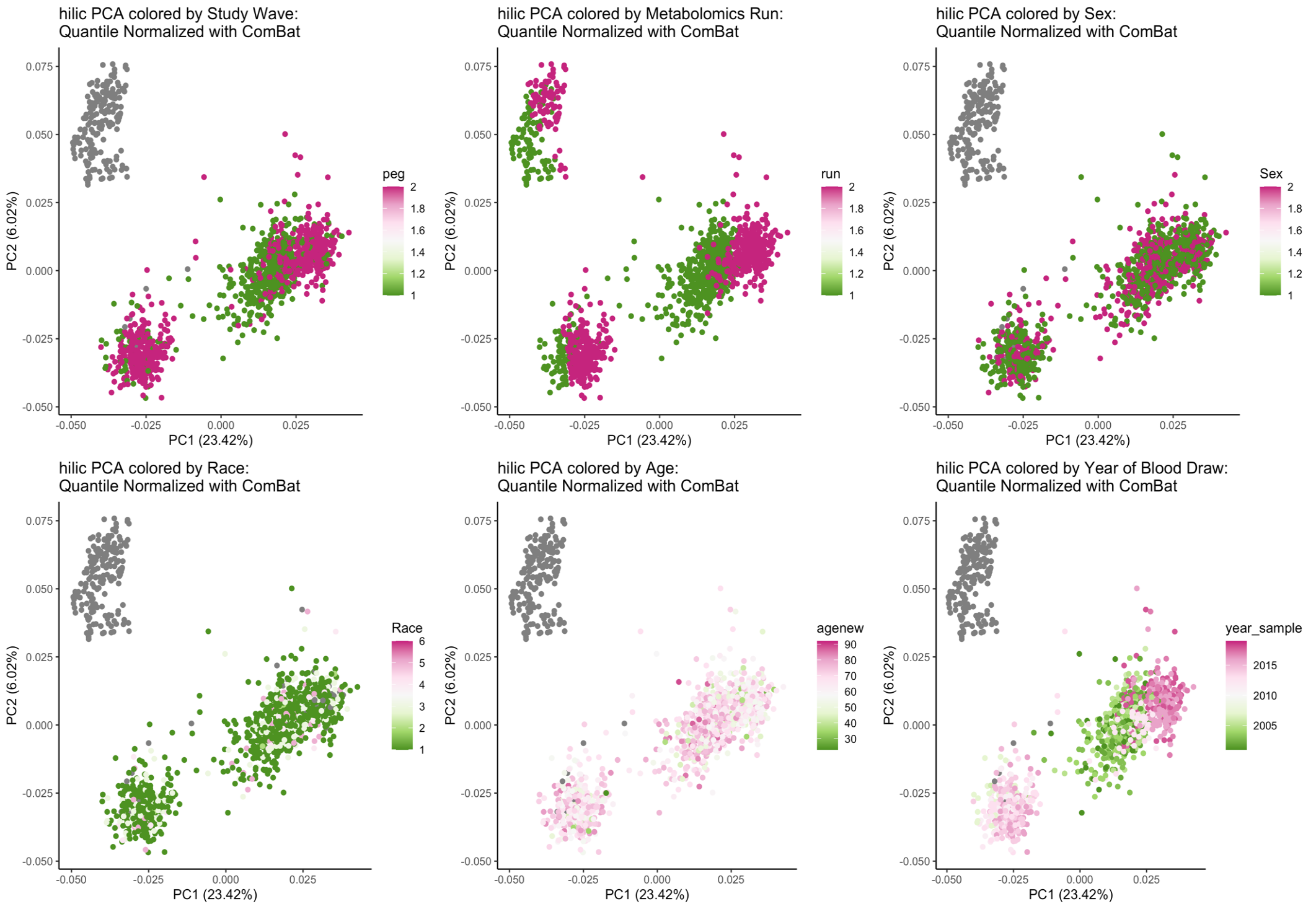
**

**Supplemental Figure 6. HILIC positive PCA of processed data, colored by different covariates.** No distinguishing variables to describe the different clusters of study samples, though there is some separation by year of sample. Note gray indicates the QC samples. Therefore, we additionally corrected for inclusion in this cluster, as variation appears technical and is very influential in MWAS (Supplemental Figure 7).
