# Supplementary material for: Untargeted serum metabolomics reveals novel metabolite associations and disruptions in amino acid and lipid metabolism in Parkinson’s disease
Source: Mol Neurodegener. 2023 Dec 19;18:100. doi: 10.1186/s13024-023-00694-5 (PMC10731845; doi:10.1186/s13024-023-00694-5)
Supplement: Supplementary file 8 — Additional file 8: Supplemental Figure 7. HILIC positive metabolomics data after processing: Log transformation, quantile normalization, ComBat batch correction, and additional adjustment for unexplained PC. [file 13024_2023_694_MOESM8_ESM.docx]

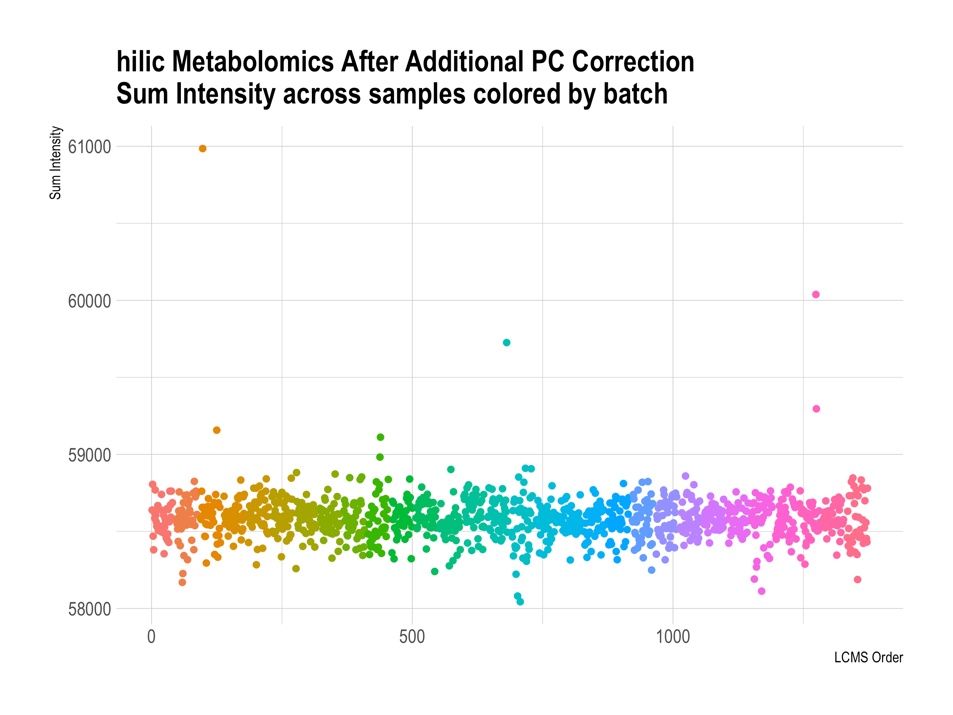

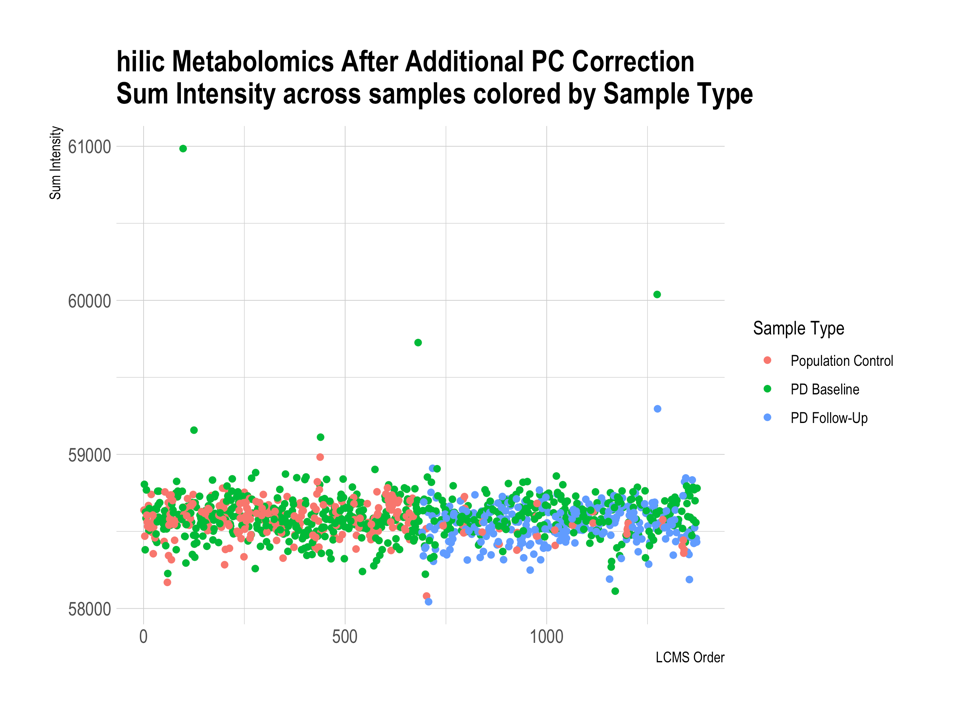


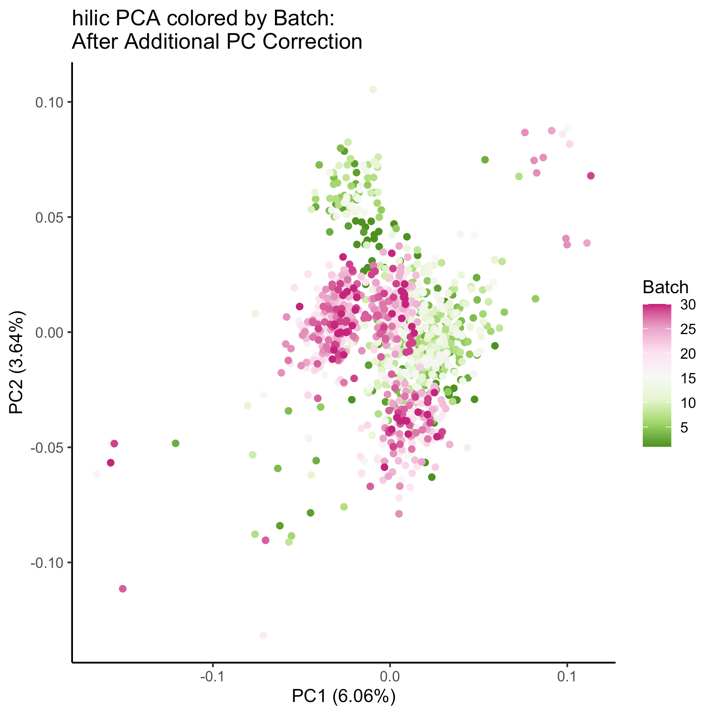

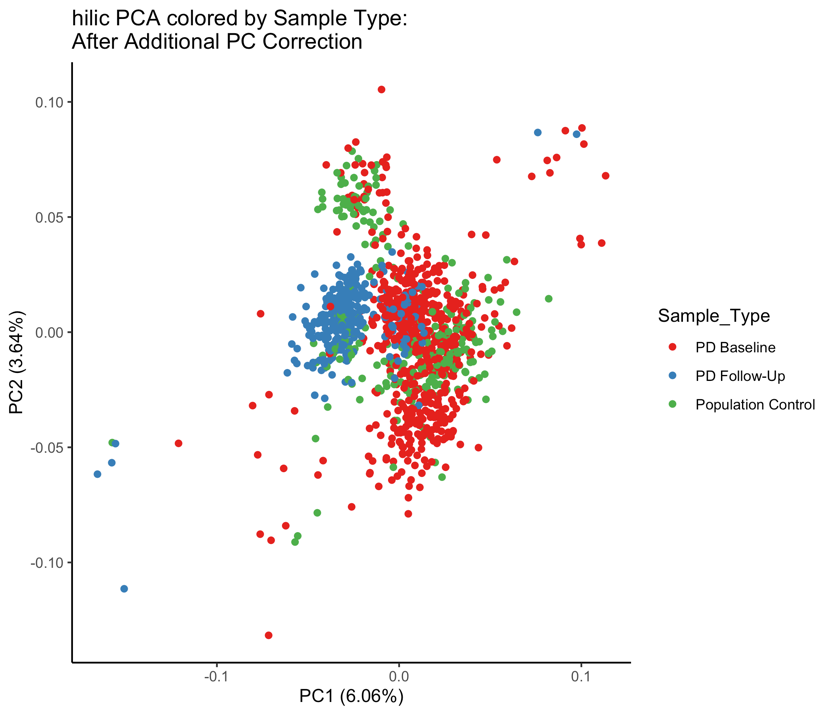


**Supplemental Figure 7. HILIC positive** **metabolomics data after processing:** Log transformation, quantile normalization, ComBat batch correction, and additional adjustment for unexplained PC.
