# Supplementary material for: Untargeted serum metabolomics reveals novel metabolite associations and disruptions in amino acid and lipid metabolism in Parkinson’s disease
Source: Mol Neurodegener. 2023 Dec 19;18:100. doi: 10.1186/s13024-023-00694-5 (PMC10731845; doi:10.1186/s13024-023-00694-5)
Supplement: Supplementary file 9 — Additional file 9: Supplemental Figure 8. Comparison of MWAS results (log2FC) when pooling the data and the processing (e.g., normalization and combat batch correction) versus processing and analyzing the data independently. (A & B) HILIC and C18 features: comparing the pooled processing logFC to a meta-analysis combining the results from each run, which was processed independently. (C & D) HILIC and C18 features: comparing the stratified results from run1 and run2, with each run was processed independently. [file 13024_2023_694_MOESM9_ESM.docx]

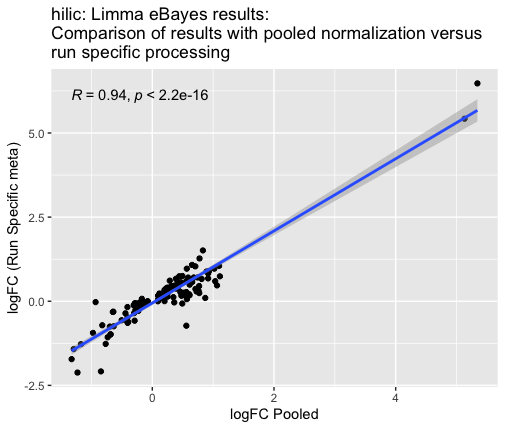

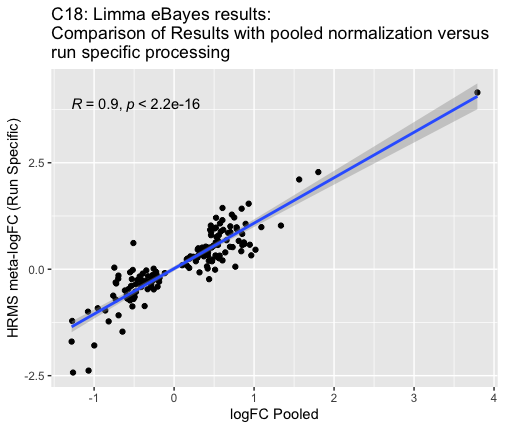


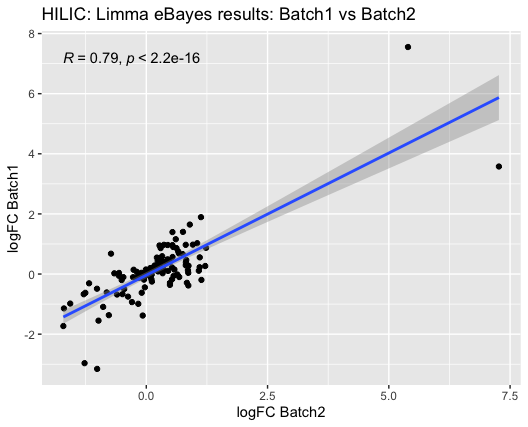

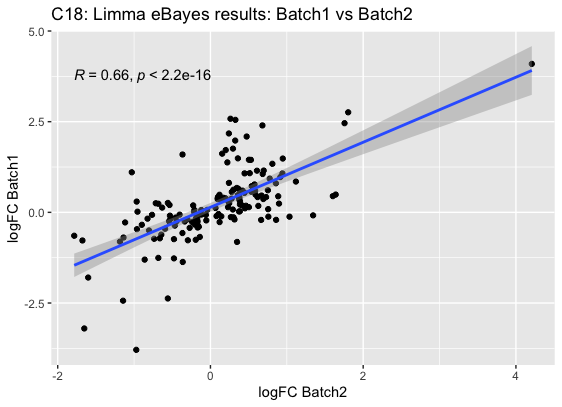


**Supplemental Figure 8**. Comparison of MWAS results (log2FC) when pooling the data and the processing (e.g., normalization and combat batch correction) versus processing and analyzing the data independently. **(A & B)** HILIC and C18 features: comparing the pooled processing logFC to a meta-analysis combining the results from each run, which was processed independently. **(C & D)** HILIC and C18 features: comparing the stratified results from run1 and run2, with each run was processed independently.
