# Supplementary material for: Untargeted serum metabolomics reveals novel metabolite associations and disruptions in amino acid and lipid metabolism in Parkinson’s disease
Source: Mol Neurodegener. 2023 Dec 19;18:100. doi: 10.1186/s13024-023-00694-5 (PMC10731845; doi:10.1186/s13024-023-00694-5)
Supplement: Supplementary file 10 — Additional file 10: Supplemental Figure 9. Volcano plots for the HILIC and C18 analysis with each run was processed (e.g., normalization and combat batch correction) and analyzed independently. [file 13024_2023_694_MOESM10_ESM.docx]

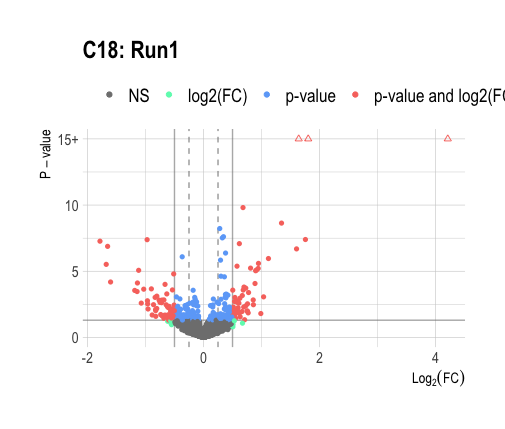

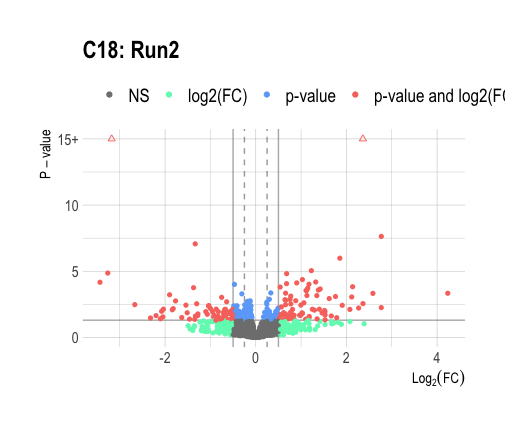


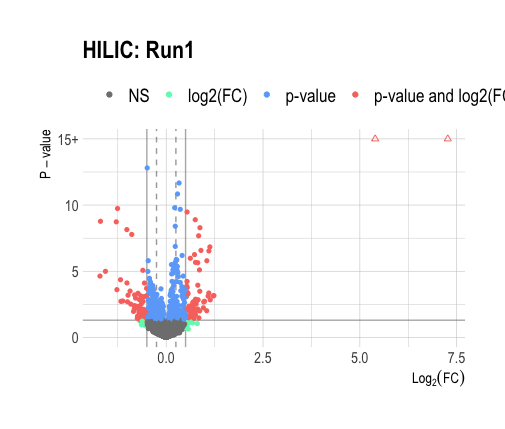

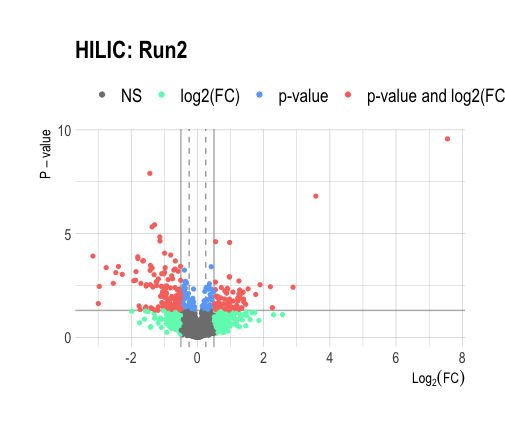


**Supplemental Figure 9**. Volcano plots for the HILIC and C18 analysis with each run was processed (e.g., normalization and combat batch correction) and analyzed independently.
