# Supplementary material for: Untargeted serum metabolomics reveals novel metabolite associations and disruptions in amino acid and lipid metabolism in Parkinson’s disease
Source: Mol Neurodegener. 2023 Dec 19;18:100. doi: 10.1186/s13024-023-00694-5 (PMC10731845; doi:10.1186/s13024-023-00694-5)
Supplement: Supplementary file 11 — Additional file 11: Supplemental Figure 10. Top metabolite results shown by HRMS run. Processing / normalization on each run independently. Mean comparisons of the crude data, shown on the log2 scale, and compared with a Wilcoxon test. Supplemental Tables 2 and 3 show results from adjusted models. [file 13024_2023_694_MOESM11_ESM.docx]

**
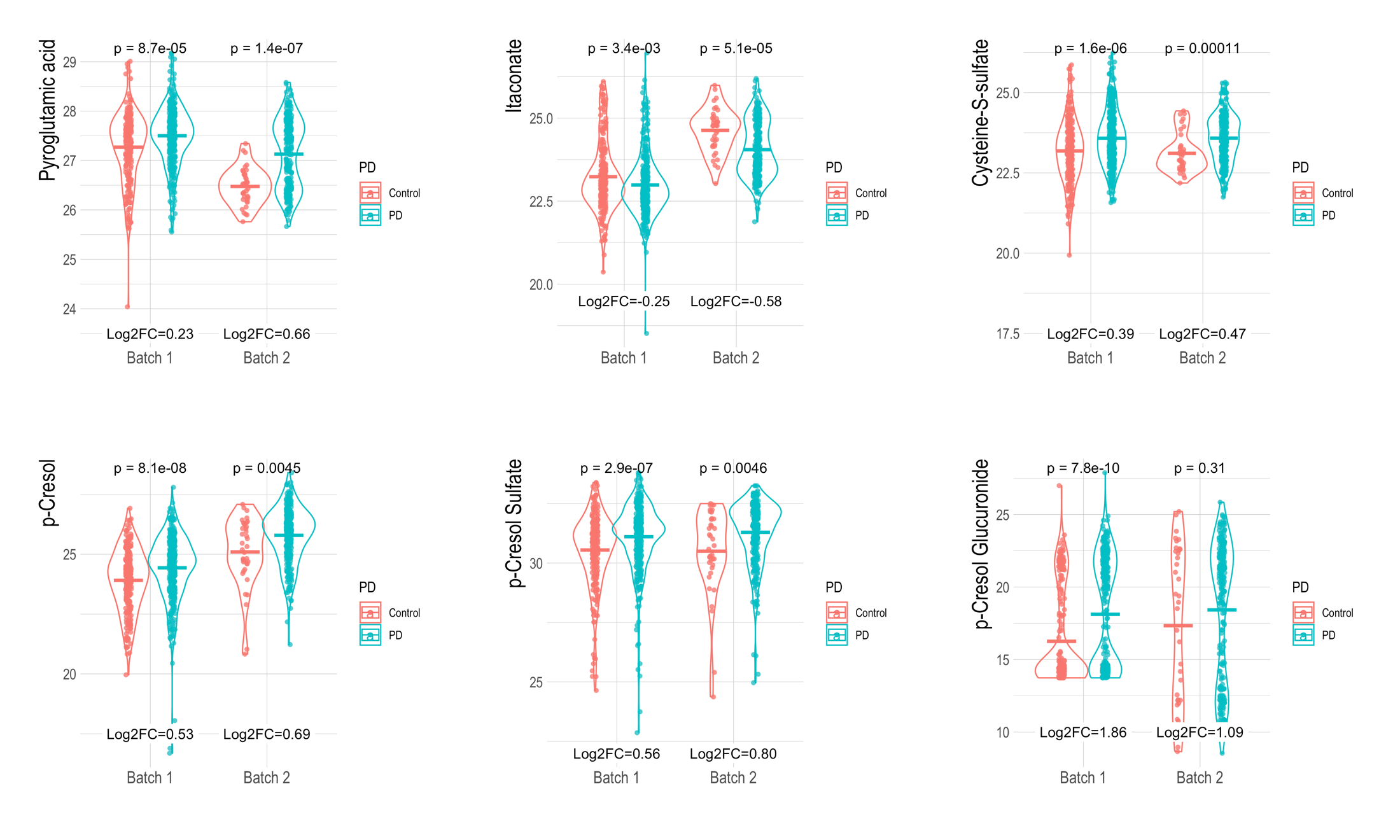
Supplemental Figure 10.** Top metabolite results shown by HRMS run. Processing / normalization on each run independently. Mean comparisons of the crude data, shown on the log2 scale, and compared with a Wilcoxon test. Supplemental Tables 2 and 3 show results from adjusted models.
